# The transcriptome of potato tuber phellogen reveals cellular functions of cork cambium and genes involved in periderm formation and maturation

**DOI:** 10.1038/s41598-019-46681-z

**Published:** 2019-07-15

**Authors:** Vijaya K. R. Vulavala, Edna Fogelman, Adi Faigenboim, Oded Shoseyov, Idit Ginzberg

**Affiliations:** 1 Institute of Plant Sciences, Agricultural Research Organization, the Volcani Center, 68 HaMaccabim Road, P. O. Box 15159, Rishon LeZion, 7505101 Israel; 2 The Robert H. Smith Institute of Plant Sciences and Genetics in Agriculture, Faculty of Agriculture, Hebrew University of Jerusalem, Rehovot, 7610001 Israel

**Keywords:** Plant molecular biology, Plant physiology

## Abstract

The periderm is a protective corky tissue that is formed through the cambial activity of phellogen cells, when the outer epidermis is damaged. Timely periderm formation is critical to prevent pathogen invasion and water loss. The outer layers of the potato periderm, the tuber skin, serves as a model to study cork development. Early in tuber development the phellogen becomes active and produces the skin. During tuber maturation it becomes inactive and the skin adheres to the tuber flesh. The characterization of potato phellogen may contribute to the management of costly agricultural problems related to incomplete skin-set and the resulting skinning injuries, and provide us with new knowledge regarding cork development in *planta*. A transcriptome of potato tuber phellogen isolated by laser capture microdissection indicated similarity to vascular cambium and the cork from trees. Highly expressed genes and transcription factors indicated that phellogen activation involves cytokinesis and gene reprograming for the establishment of a dedifferentiation state; whereas inactivation is characterized by activity of genes that direct organ identity in meristem and cell-wall modifications. The expression of selected genes was analyzed using qPCR in native and wound periderm at distinct developmental stages. This allowed the identification of genes involved in periderm formation and maturation.

## Introduction

Potato tubers are covered with a protective corky tissue called the periderm. The periderm is made up of three types of cells^1^. The outer layers, the potato skin, are composed of suberized phellem cells. The inner layers, known as the phelloderm, are made up of parenchyma-like cells. In between the phellem and the phelloderm, there is a single-cell, meristematic layer of secondary origin called the phellogen or cork cambium. Outward cell divisions of the phellogen form the skin and inward divisions form the phelloderm. Following tuberization, the original epidermis is replaced by the protective periderm; subepidermal cells undergo dedifferentiation to initiate the phellogen cells that sequentially initiate skin formation. This developmental stage will be referred to here as phellogen activation/skin initiation. The phellogen remains active and adds more skin cells to the expanding tuber — this developmental stage is named immature skin or immature periderm. In immature skin, the actively dividing phellogen is labile and prone to fracture, allowing the separation of the skin from the underlying phelloderm and tuber flesh, and resulting in the costly agricultural problem of skinning injuries^2,3^. At the end of the period of growth, the tuber ceases to expand, no new skin cells are required and the phellogen undergoes inactivation. As a result, skin layers adhere strongly to the tuber flesh — a processes known as skin-set or skin/periderm maturation. The cellular mechanisms of phellogen activation and inactivation and the respective skin formation and maturation are as yet unknown.

Studies on periderm development in potato can be done by inducing the formation of wound periderm via the removal of the tuber skin or by excising discs of tuber flesh using a cork borer, and then allowing the exposed tissue to heal (procedures analogous to harvest of *amadia*/reproductive cork^4^). Phellogen initials are first noticeable at around 3–5 days after wounding^5–7^. This type of periderm is similar to the native periderm in terms of tissue origin, structure and morphology, although its chemical composition differ^8–12^.

It has been suggested that cytological events that lead to potato phellogen initiation and periderm development may follow a transient increase in auxin and lipid hydroxyperoxide levels, both peaking at 20 to 30 min after the wounding of a potato tuber^13^. This is followed by mitotic activity of cells competent to produce periderm, starting from 120 min post-wounding^13^. In accordance, the expression of cell-cycle genes was detected^7^. Cell divisions were accompanied by an increase in polymerized actin and microfilament bundles in cells at the wounding site^14^.

Studies on periderm ontogeny in woody species suggested that cork develops following an increase in auxin levels that promotes ethylene production, which, in turn, is a major activator for phellogen initiation^15^. It has been suggested that the GRAS transcription factor *SHORT-ROOT 2B* (*SHR2B*), which determines the specification of stem cell niche and radial patterning, may play a role in the phellogen of cork oak (*Quercus suber*) and *Populus*^16,17^. It was further suggested that the factor might act through the modulation of cytokinin homeostasis^17^.

As to additional genes that may be expressed in the phellogen and its phellem progeny, Boher *et al*. identified a suberin-associated feruloyl transferase (*FHT*) whose expression and accumulation within the potato periderm is restricted to phellogen-derived cells with phellem identity^18^. Tissue wounding has been found to induce the expression of *FHT* and the protein accumulates during the healing process.

A transcriptomic approach has also been used to profile cork oak phellogen^19,20^ and phellem from oak and *Populus*^21,22^, in order to study cork quality development. Good-quality cork was found to be enriched with stress-related genes, the synthesis of suberin and lignin was promoted, and hormonal regulation involved ABA, ethylene and auxins. In contrast, bad-quality cork showed a consistent up-regulation of genes belonging to the flavonoid pathway, as well as different modulation of cell-wall genes, resulting in a thinner cork layer^19,20^. Several genes that may be involved in regulating the phellogen/cork cambium have been discussed^21,22^. These include chromatin-remodeling genes, *FLOWERING LOCUS C* (*FLC*), which is involved in xylem differentiation, and some transcription factors that are related to both stem-cell maintenance in cambium and stress responses^21^.

Apart from characterizing the cambial activity of the potato phellogen in the formation of corky phellem, the phellogen in potato also has significant agricultural importance. Several physiological disorders of potato skin may be related to phellogen activity. These include incomplete skin-set, skinning injuries, skin russeting and loss of red pigmentation in wounded periderm. The characterization of phellogen activation/inactivation “switch” may contribute to the improved management of these costly problems.

In order to characterize phellogen activity, a transcriptome was prepared from isolated potato phellogen cells obtained by laser capture microdissection (LCM). Highly expressed genes indicated high levels of cell proliferation and confirmed the similarity of potato phellogen to the vascular cambium at a molecular level. Selected candidate genes exhibited differential and high expression at the phellogen initiation/skin formation stage or the phellogen inactivation/skin maturation stage.

## Results

### Visualization of phellogen cells

**Figure 1:**
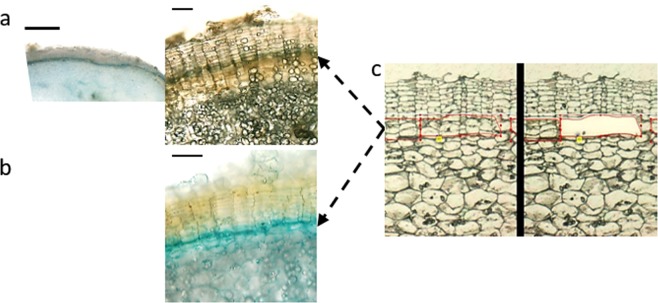
Visualization of the phellogen layer. A GUS reporter assay (**a**,**b**) was used to examine the phellogen layer. Microtubers were induced from transgenic potato plants carrying *GUS* fused to (**a**) the promoter region of the *MITOTIC CYCLIN B1* gene or (**b**) the Arabidopsis cytokinin responsive element *ARR5*, and sections of microtubers were subjected to GUS (5-bromo-4-chloro-3-indolyl-β-D-glucuronide) staining. Phellogen appears as blue layer (black arrows). Laser capture microdissection was used to isolate the phellogen cells (**c**, outlined in red) – left panel, before excision of the phellogen; right panel after the excision. Potato skin is characterized by well-organized columns of phellem cells. The phellogen layer is located just below the phellem, each cambial cell at the base of a phellem column. The actively dividing phellogen cells can be easily identified by their puffy morphology (**c**). Thin bars: 100 µm; thick bar: 1000 µm.

To visualize the phellogen in developing periderm, cultures of *in vitro*-induced microtubers were prepared from transgenic plants carrying constructs with the cell cycle marker *CycB1*^23^ (Fig. 1a) and the cytokinin-responsive element *ARR5*^24^ (Fig. 1b), both fused to *GUS* reporter gene. The *ARR5* was chosen based on previous suggestion of cytokinin modulation of *Populus* phellogen activity^17^. Free-hand sections of 8- to 10-day-old microtubers were examined under a binocular following GUS staining. The phellogen appeared as a blue one-cell layer with well-organized columns of flattened phellem cells above it, and columns of parenchyma-like phelloderm cells that were less organized below it.

For preparation of the phellogen-specific transcriptome, the challenge was to isolate the phellogen cells in their native form with minimum contamination of the surrounding phellem or phelloderm cells. This was done by LCM following the identification of the actively dividing phellogen cells with puffy morphology, as detailed in ‘Materials and Methods’ and demonstrated in Fig. 1c.

### Characterization of the phellogen transcriptome

For preparation of the transcriptome, RNA was extracted from LCM-isolated phellogen cells and subjected to RNA sequencing. Data (about 140 million reads) were mapped against the potato genome; only 70% of the reads were mapped. This resulted with 26,335 contigs of which, around 10,000 were annotated genes, and the rest were contigs with no defined gene annotation in the potato genome database (annotated as CUFF in Table S1 column A). Contigs smaller than 100 bp that were not mapped to the genome and could not be verified as “true” genes, were removed from the data. The final list consisted of 6,754 genes/contigs. To improve the annotation, BLAST analyses were performed against potato, tomato and Arabidopsis CDS sequences using Sol Genomics Network and TAIR databases, respectively; 4,066 genes had a putative known function, while 2,688 genes were of unknown function (Table S1).

**Figure 2:**
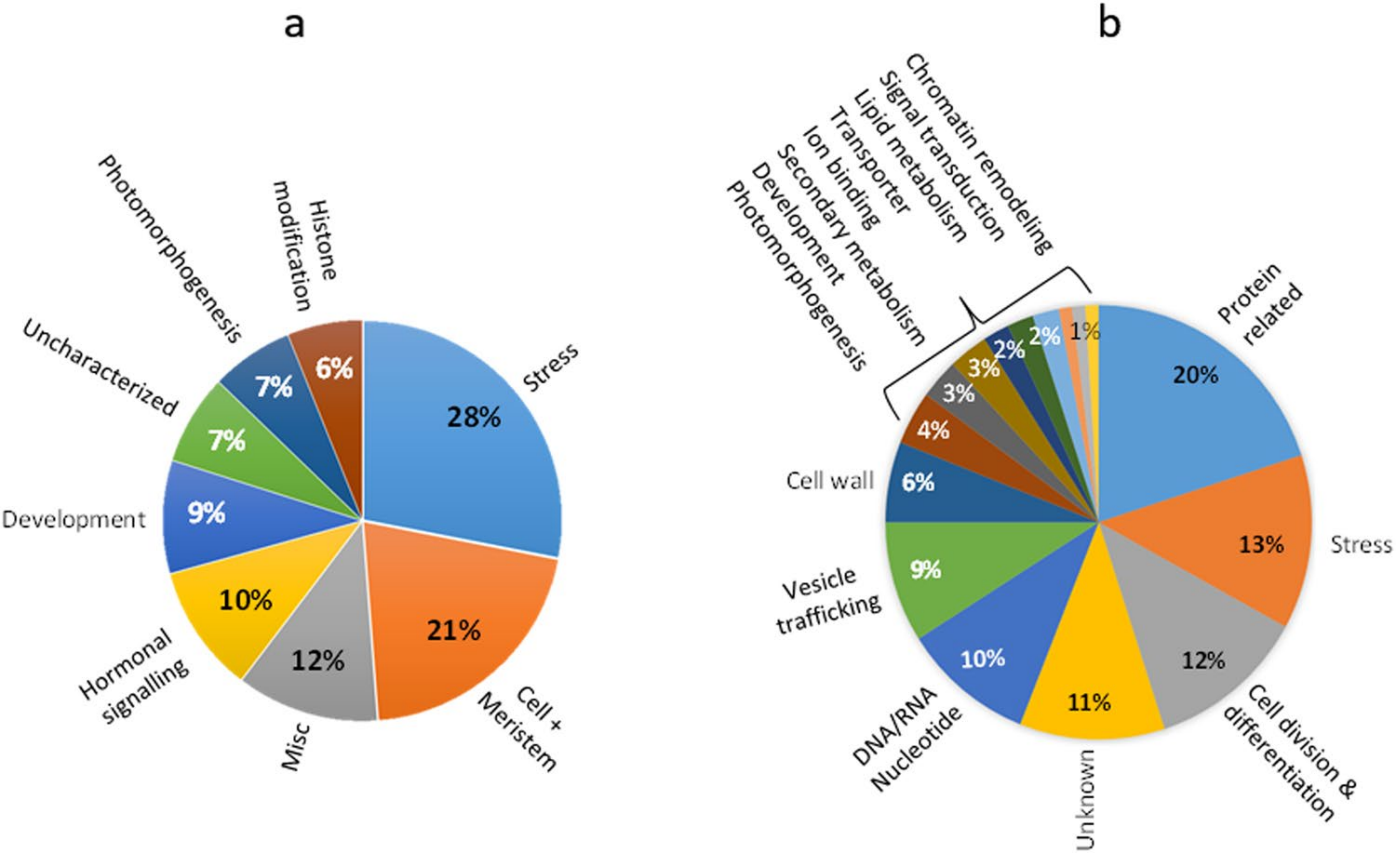
Functional analysis of all transcription factors found in the phellogen transcriptome (**a**) and of the 100 most highly expressed transcripts (**b**). Transcripts were manually annotated to their roles in the plant based on BLAST studies and a literature survey, and then categorized into functional groups (Table S1 column V). The charts show the relative proportion (in percentage) of the transcripts in each group.

All genes coding for transcription factors in the transcriptome, and a group of the 100 most highly expressed genes were further annotated for better categorization of their specific functions in the phellogen using a literature survey of their orthologs from Arabidopsis (Table S1 column V). A total of 164 transcription factors were found whose functions may demonstrate the cellular activities of the phellogen. Most of the regulatory functions were related to the processes of cell division and differentiation (43%), including cellular and meristematic activity, developmental processes, photomorphogenesis and histone modification (Fig. 2a). Others (29%) regulated functions related to hormonal signaling or miscellaneous and uncharacterized functions. Another major group of transcription factors (28%) was associated with the regulation of stress-related functions.

As for the 100 genes that were expressed at the highest levels, most of the identified functions were related to proliferative activity and differentiation (Fig. 2b), including protein-related functions, mainly ribosomal proteins (20%); functions related to cell division and differentiation (12%); DNA- and RNA-related processes (10%); vesicle trafficking (9%); the cell wall (6%); photomorphogenesis (4%); secondary metabolism (3%); ion binding (2%); lipid metabolism (2%) and signal transduction (2%). The remaining genes were stress-related (13%). Application of MapMan software to these stress-related functions revealed highly expressed redox-related processes in the phellogen, as well as heat-shock proteins and proteolysis-related activities (Fig. S1). The overall distribution of functional categories of the highly expressed genes in the phellogen was in accordance with the findings of the previous analysis of transcription factor-regulated processes.

### Phellogen meristematic activity

Further analyses of the transcriptome were aimed at searching for known meristematic genes and deciphering what type of meristem the phellogen is. A literature survey allowed the listing of genes related to shoot apical, root apical, floral and vascular cambium meristems. Of all of the meristem-related genes that were found in the phellogen transcriptome, the majority (62%) were related to the vascular cambium, 14% were related to the shoot apical meristem, 14% were related to the floral meristem and 10% were related to the root apical meristem (Table S2).

To evaluate the specificity of the potato phellogen transcriptome and its relation to that of the cork cambium, we compared our data with the cork oak transcriptome. Recently, Boher *et al*. reported the *Q*. *suber* transcriptome following isolation of its cork consisting of phellem and phellogen cells^21^. About 40% (2752) of the annotated genes in the transcriptome of the potato phellogen were shared with those of the cork oak (Table S1, Column J). Sixty-two of the potato and cork oak orthologous genes fell within the group of the 100 most highly expressed phellogen transcripts described above. Categorization of these cork oak orthologs indicated activity related to ribosomal proteins (19%), cell division and differentiation (19%), vesicle trafficking (17%), mRNA-related processes (15%), signal transduction (8%), the cell wall (6%), proteolysis (6%) and stress (10%). These data are in accordance with the analysis done for potato phellogen (Fig. 2b) and demonstrate the cellular processes that occur during phellogen activity in *planta*.

### Anatomical study of potato phellogen activity and periderm development

**Figure 3:**
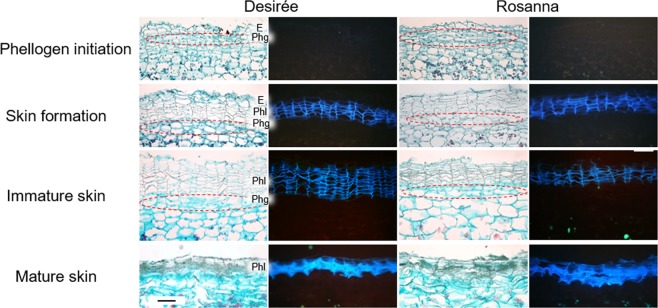
Developmental stages of native periderm of potato cultivars Desirée and Rosanna. Tubers were collected following tuberization and through maturation. Tissue samples were taken from their surface and embedded in paraplast. Cross-sections were made, stained with Safranin/Fast green and viewed under a light microscope (left panel) and a UV microscope (right panel, black background), to examine tissue morphology and the autofluorescence of suberized cells, respectively. At around 3 weeks after sprout emergence (WAE), phellogen initials (circled) appeared below the early tuber epidermis. A few days later (4 WAE), the first layers of suberized phellem could be seen above the phellogen and below the epidermis, indicating skin formation. Note the characteristic morphology of the phellem as columns of flattened cells that autofluoresce under UV light. Maximal phellem/skin cell formation occurred at around 8 WAE, when the skin was at its immature stage. When tuber expansion ends (11–12 WAE), phellogen activity ceases, and the skin matures as evident by the compactness of the phellem layers. E – epidermal cell layer, Phg – phellogen layer, Phl – phellem cells. Bar: 100 µm.

To analyze the expression profiles of phellogen-related genes, it was necessary to define phellogen developmental stages and to sample the periderm at those defined stages. For that purpose, an extensive anatomical study was conducted of tuber surface collected every week starting at 3 weeks after sprout emergence (WAE) (tuberization stage) and continuing through 12 WAE (periderm/skin maturation). Tissue blocks for anatomical study and periderm peels for gene-expression analysis were collected in parallel. Tissue samples were observed under a light microscope and a UV microscope and four developmental stages were defined (Fig. 3): (a) phellogen/skin initial stage (3 WAE), when phellogen initials appear below the early tuber epidermis; (b) skin formation (4 WAE), when the first layers of suberized phellem can be seen above the phellogen and below the epidermis; (c) immature skin (8 WAE), when the phellogen is at its apparent maximal activity and skin phellem layers are being added at the most rapid rate, and (d) skin maturation (12 WAE), at which point the phellogen is inactivated, resulting in the skin-set process. These stages were demonstrated similarly for cv. Desirée and Rosanna (Fig. 3); the latter cultivar was chosen as it frequently exhibits severe skinning problems under local growth conditions.

### Linking gene functions to phellogen activation and inactivation

Seventy three genes were selected based on the following criteria: (a) a strong signal in the transcriptome, (b) being an optional member of a suberin-related gene family, (c) being hypothesized to be involved in cell division and differentiation and (d) being involved in the regulation of cork cambium (genes are listed in Table S1, Column W). To verify high and differential expression of the genes in developing potato periderm, PCR analysis was conducted to compare immature and mature potato periderm with tuber flesh and leaf tissue (Fig. S2a).

**Figure 4:**
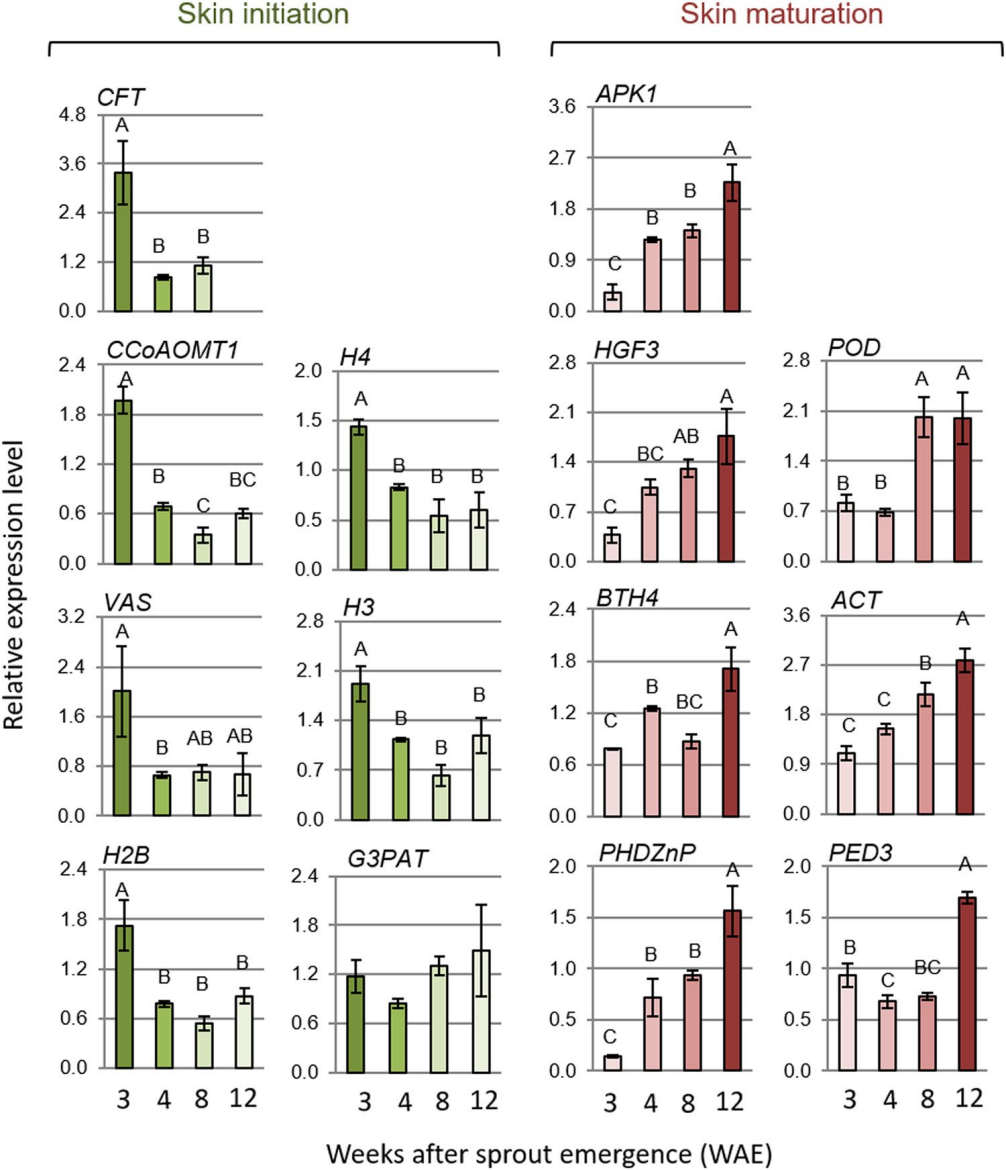
Expression profile of phellogen-related genes at four stages of periderm development. Periderm peels were collected from cv. Desirée tubers: at phellogen/skin initiation (3 WAE), at skin formation (4 WAE), from immature skin (8 WAE) and from mature skin following skin-set (12 WAE). Skin anatomy at the respective stages is illustrated in Fig. 3. Transcript levels were monitored by qPCR and expression levels were normalized relative to the level of the reference gene *α*-*NAC*. Genes exhibiting differential and high expression at skin initiation are presented on the left side of the figure and genes expressed mainly in the mature skin are presented on the right. Values represent an average of three biological replicates with SE bars. Statistically significant differences between means were identified using Student’s *t*-test; different letters indicate significantly different values (*P* < 0.05).

Of the 73 genes, 39 exhibited differential and high expression in immature or mature periderm and low or no expression in leaves and tuber flesh, and were further analyzed. This time, the expression of the selected 39 genes was tested at the four stages of phellogen activity described above, using qPCR for accurate gene profiling (Fig. S2b). This process allowed us to identify 14 genes that were associated with phellogen activity. Seven genes were highly and differentially expressed at the phellogen-initiation stage (Fig. 4, left): *GDSL LIPASE-LIKE* (*CFT*, Sotub01g036860); *CAFFEOYL COENZYME A O-METHYLTRANSFERASE 1* (*CCoAOMT1*, Sotub02g031720); *VASCULAR TISSUE SIZE* (*VAS*, Sotub01g040060); the histone protein-related genes *H2B* (Sotub03g016600), *H3* (Sotub10g009520) and *H4* (Sotub11g029670); and *GLYCEROL-3-PHOSPHATE SN-2-ACYLTRANSFERASE 3* (*GPAT3*, Sotub01g032090). Seven genes were highly and differentially expressed following phellogen inactivation (skin maturation) (Fig. 4, right): *PHD-FINGER FAMILY HOMEODOMAIN PROTEIN/ HAT3*.*1* (*PHDZnP/HAT3*, Sotub01g044570), *ACTIN 7* (*ACT7*, Sotub03g020330), *NON-RECOGNITION-OF-BTH 4/ MEDIATOR 15* (*BTH4/MED15*, Sotub04g009440), *PEROXIDASE 49-LIKE* (*POD*, Sotub02g027930), *PEROXISOMAL DEFECTIVE 3/COMATOSE* (*PED3/CTS*, Sotub04g020700), *PROTEIN KINASE* (*APK1/AtATH8*, Sotub04g014120) and *ENHANCER OF AG-4 PROTEIN 2* (*HGF3/HUA2*, Sotub02g005440). The expression of those 14 selected genes was also examined in the periderm of cv. Rosanna, which is prone to skinning injuries (Fig. S3), and were found to be similar to those observed for cv. Desirée.

**Figure 5:**
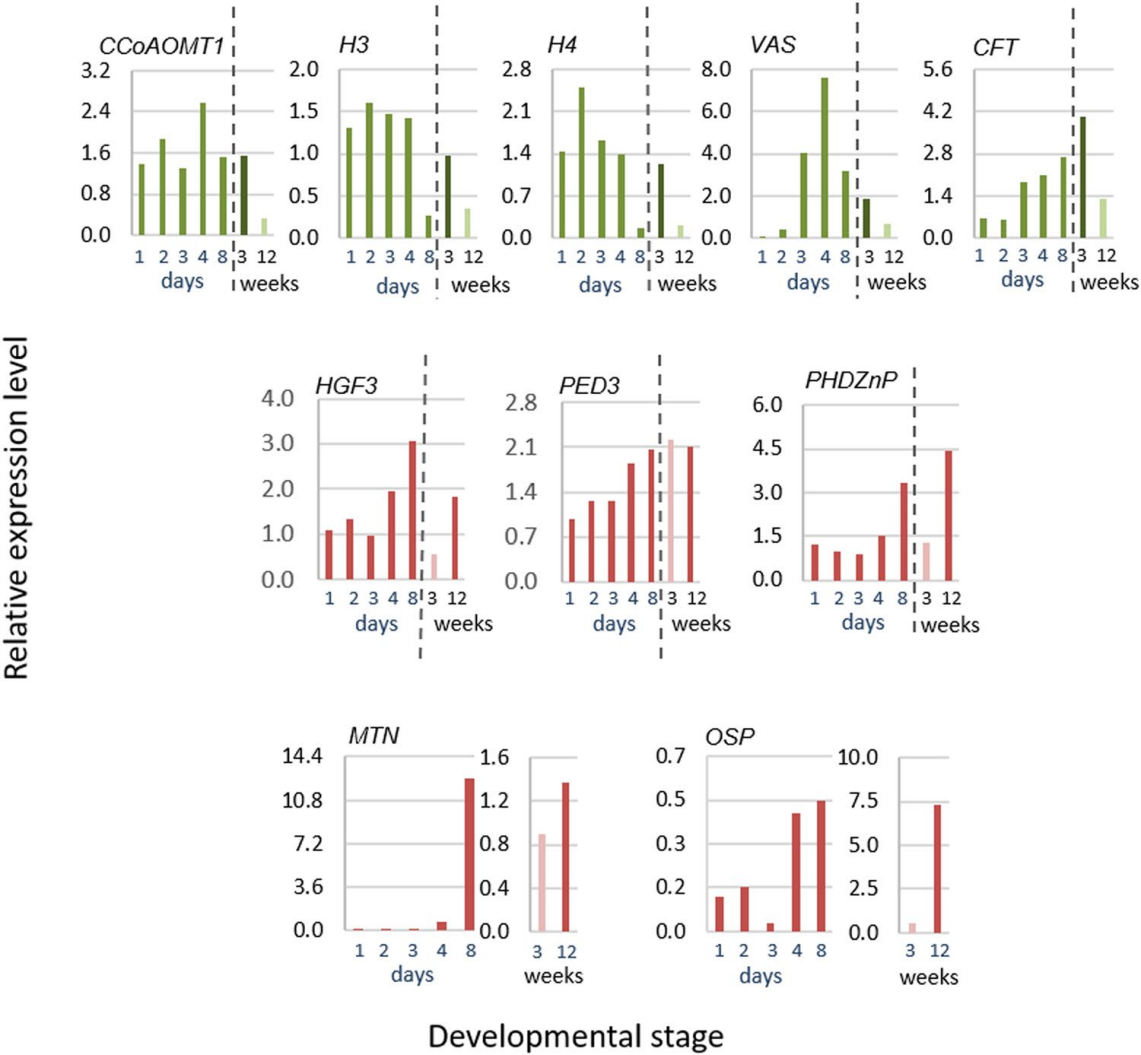
Expression profile of phellogen-related genes in developing wound periderm. Wound periderm develops at the surface of potato tuber slices kept in the dark and under high humidity. The surface of the slices was collected at 1-d intervals after wounding. Between Day 1 and Day 3, wounding induced the formation of a closing layer and phellogen initials formed below it. On Day 3, columns of new phellem cells were added below the closing layer, and from Day 4 on, the newly formed phellem underwent suberization. The wound periderm was mature on Day 8. A detailed anatomical description is provided in Fig. S4. Gene expression during wound healing (monitored in days) was compared to that observed during two stages of native periderm development (monitored in weeks): phellogen initial stage at 3 weeks after sprout emergence (WAE) and mature skin at 12 WAE. Transcript levels were monitored by qPCR and expression levels were normalized relative to the level of the reference gene *α*-*NAC*.

To further examine gene expression during the distinct stages of phellogen activity, we used the model system of wound periderm that exhibits similar stages of phellogen activity as the native periderm (Fig. S4). *CCoAOMT1*, *VAS*, *H4* and *H3*, which were shown to associate mainly with the early stages of phellogen initiation, were also up-regulated during wound periderm induction, and their levels were reduced upon wound periderm maturation and wound closure (Fig. 5). An exception to this pattern was *CFT*, which exhibited increased expression during the development of wound periderm, but was related to phellogen initiation in the native periderm. *PED3/CTS*, *PHDZnP/HAT3* and *HGF3/HUA2*, which were shown to associate mainly with phellogen inactivation (skin maturation), exhibited high levels of expression during the maturation of wound periderm as well (Fig. 5). Two additional genes that were not found in the phellogen transcriptome were included in this experiment: *METALLOTHIONEIN 2B* (*MTN*, Sotub06g010040) and *ORGAN-SPECIFIC PROTEIN S2* (*OSP*, Sotub07g011080). We use these genes as markers for the skin-set process and they were used here to validate the developmental stage of wound closure. Their elevated expression was found to be in accordance with native- and wound-periderm maturation, as demonstrated in Fig. 5. Overall, the data strongly link the selected genes to the critical stages of phellogen activation/skin formation and phellogen inactivation/skin maturation

### Protein–protein association networks within the phellogen transcriptome

**Figure 6:**
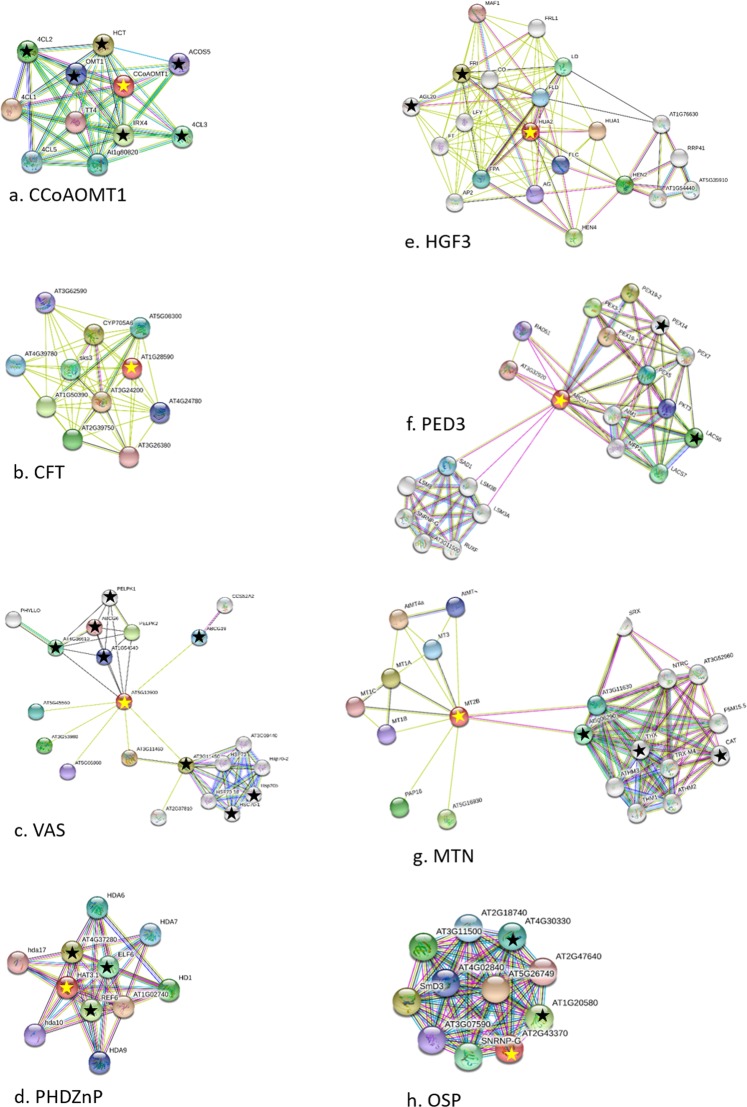
STRING functional protein association networks (https://string-db.org/) of phellogen-related genes. Arabidopsis orthologs of the potato genes were identified and their TAIR IDs were used in the STRING search engine^65^. For each gene cluster, the query phellogen gene is marked with a yellow star and putative associated proteins that were also found in the potato phellogen transcriptome are marked with a black star. Data on the members of the interactomes is given in Table S1, Column Y, and in Table 1. In brief, (**a**) the interactome of CCoAOMT1 included phellogen-related genes, which are all involved in early stages of lignin biosynthesis. (**b**) The interactome of CFT included proteins of the GDSL esterase/lipase group that play a role in suberization, but none of those proteins were found in the phellogen transcriptome. (**c**) The interactome of VAS included phellogen proteins whose activities are related to heat stress-response and chromatin regulation, a cell-cycle regulator involved in the maintenance of shoot apical meristem, and ATP-binding cassettes which are involved in cell-wall synthesis. (**d**) The interactome of PHDZnP/HAT3 included the phellogen-related genes which are involved in the chromatin remodelling that controls meristem activity and organ polarity in an already established meristem. (**e**) The interactome of HGF3/HUA2 included the phellogen-related genes which are involved in specification of organ identity in meristem. (**f**) The interactome of PED3/CTS included the phellogen-related genes which are involved in transport and beta-oxidation of fatty acids and putatively with suberization. (**g**) The interactome of MTN included phellogen-related redox activities. (**h**) The interactome of OSP included phellogen-related small ribonuclear proteins that are involved in splicing of pre-mRNA and are required for plant development.

The STRING protein–protein interaction network (version 10.5; https://string-db.org/) was used to analyze putative protein association networks within the phellogen transcriptome data using the IDs of their orthologs from Arabidopsis. Then we back-searched for the protein members of the resulting interactomes in the phellogen transcriptome, to identify associations between the selected genes and additional phellogen functions. For the following see Fig. 6, Table 1, and Table S1, Column Y. The interactome of phellogen initiation-related protein CCoAOMT1 included proteins involved in early stages of lignin biosynthesis. The interactome of CFT included GDSL esterase/lipase activities that may be putatively involved in suberin biosynthesis^25^, however none of these CFT-interactome members were found in the phellogen transcriptome. The VAS interactome was an assembly of three phellogen-related groups. One included ATP-binding cassettes, which are involved in cross-membrane transport of cutin/suberin-related fatty acids^25–27^. The second included heat shock-responsive proteins which are involved in developmental processes such as meristem maintenance, floral development, and plant-size maintenance. The third group had only one member, a cell-cycle regulator involved in the maintenance of shoot apical meristem. The interactome of PHDZnP/HAT3 included the phellogen-related activities of histone modifications and chromatin remodelling that are important functions for development and differentiation. The MTN interactome included phellogen-related redox reactions, and the interactome of OSP included phellogen-related small ribonuclear proteins, which are involved in the splicing of pre-mRNA and are required for plant development. Additional interactomes are presented in Fig. S5 and Table S3. The H2B interactome involved in transcription regulation, post-translational modifications of histones and nucleosome remodelling, and is important for development and differentiation. The histone proteins H3 and H4 interactomes overlapped with one another considerably; whereas H3 included phellogen-related additional histone protein H2A6; and H4 interactome included the complexes involved in nucleosome remodelling and transcriptional regulation. The GPAT3 interactome interacted with glycerolipid and lysophosphatidic acid metabolism required for lipid synthesis for cell membrane. The interactome of ACT7 included the phellogen-related proteins involved in actin filament elongation during cell divisions. The interactome of BTH4/MED15 contained proteins involved in transcriptional regulation, but no such proteins were found in the phellogen transcriptome. The POD interactome contained phellogen-related activities that are involved in lignin biosynthesis. The interactome of PED3/CTS included the phellogen-related proteins that are involved in beta-oxidation of fatty acids and controls the intracellular transport of proteins to the peroxisome, an important function in suberin biosynthesis. Overall the results of the protein-association analysis confirmed the selected phellogen/skin-initiation and maturation-related gene functions associated with cell differentiation, meristematic maintenance and the biosynthesis of cell-wall components. This will be discussed below.

**Table 1:** Functional protein association networks within the phellogen transcriptome

Phellogen query	Query annotation	Members of interactome	TAIR ID	Protein function
CCoAOMT1 (AT4G34050)	Caffeoyl coenzyme A; *O*-methyltransferase 1; lignin biosynthesis	4CL2 (4-coumarate-CoA ligase 2)	AT3G21240.1	Lignin biosynthesis
		4CL3 (4-coumarate-CoA ligase 3)	AT1G65060.1	Lignin biosynthesis
		ACOS5 (acyl-CoA synthetase 5)	AT1G62940.1	Lignin biosynthesis
		HCT (hydroxycinnamoyl transferase)	AT5G48930.1	Lignin biosynthesis
		IRX4 (cinnamoyl CoA reductase)	AT1G15950.1	Lignin biosynthesis
		OMT1 (*O*-methyltransferase 1)	AT5G54160.1	Lignin biosynthesis
VAS (AT5G13900)	Lipid transfer-like protein; promotion of pro-cambial and pericycle cell division	Hydrolase, alpha/beta fold family protein	AT4G36610.1	Cross-membrane transport of fatty acids
		ATZRF1A (zuotin-related factor A1)	AT3G11450.1	Meristem maintenance
		NHL11 (late embryogenesis abundant hydroxyproline-rich glycoprotein)	AT1G54540.1	Embryogenesis-related
		ABCG6 (ATP-binding cassette G6)	AT5G13580.1	Cross-membrane transport of fatty acids
		ABCG19 (ATP-binding cassette G19)	AT3G55130.1	Cross-membrane transport of fatty acids
		HSP70B (heat-shock protein 70 kDa B)	AT1G16030.1	Stress
		HSC70-1 (heat-shock protein 70 kDa 1)	AT5G02500.1	Stress
		PELPK1; PRO-GLU-LEU|ILE|VAL-PRO-LYS 1	AT5G09530.1	Positive regulator of germination
CFT (AT1G28590)	GDSL esterase/lipase	NA	NA	NA
PHDZnP (AT3G19510)	Homeobox protein HAT3.1; chromatin remodelling	REF6 (relative of early flowering 6)	AT3G48430.1	Chromatin remodeling /cell elongation
		ELF6 (EARLY FLOWERING 6)	AT5G04240.1	Chromatin remodeling /cell elongation
		MRG1 (MORF RELATED GENE 1)	AT4G37280.1	Chromatin remodeling
HGF3/HUA2 (AT5G23150)	ENHANCER OF AG-4 2; specification of organ identity in meristem	FRI (FRIGIDA)	AT4G00650.1	Flowering time regulation
		AGL20 (AGAMOUS-like 20)	AT2G45660.1	Organ identity in meristem
PED3/CTS (AT4G39850)	Beta oxidation of fatty acids	LACS6 (long-chain acyl-CoA synthetase 6)	AT3G05970.1	Beta oxidation of fatty acids
		PEX14 (peroxin 14)	AT5G62810.1	Beta oxidation of fatty acids
MTN (AT5G02380)\	Metallothionein 2B; metal chelator of copper and zinc	THX (thioredoxin X)	AT1G50320.1	Redox homeostasis
		CAT (catalase 2)	AT4G35090.1	Redox homeostasis
		2-CYS PEROXIREDOXIN B	AT5G06290.1	Redox homeostasis
OSP (AT2G43370)	RNA-binding (RRM/RBD/RNP motifs) family protein	Small nuclear ribonucleoprotein-like protein	AT1G20580.1	Splicing of pre-mRNA
		Small nuclear ribonucleoprotein E	AT4G30330.1	Splicing of pre-mRNA

Analysis was performed using the STRING program (https://string-db.org/) based on Arabidopsis orthologs of phellogen-selected genes. This table lists interactomes based on Fig. 6.

## Discussion

### Phellogen characteristics and its shared functions with cork and vascular cambium

Despite their importance *in planta*, there is lack of information regarding the characteristics of phellogen cells with respect to their initiation, proliferative activity and their inactivation following the completion of periderm development. The potato periderm and the cork of *Q*. *suber* are the accepted model to study cork development; only recently the periderm development from the pericycle of root and hypocotyl of Arabidopsis was described^28^.

Transgenic potato plants expressing reporter constructs of *CycB1*^29,30^ and the cytokinin-responsive regulator *ARR5*^24^ demonstrated the cell-division activity of the tuber phellogen and indicated the involvement of cytokinin (Fig. 1). Similarly, the regulation of the phellogen from *Populus* was also suggested to involve the modulation of cytokinin homeostasis^17^. The transcriptome of potato phellogen includes genes that determine specific aspects of stem cell niche and radial patterning such as *SCRAMBLED* (*SCM*^31^), *RING1B*^32^, *TOPLESS-RELATED 2* (TPR-2^33^), *NO APICAL MERISTEM* (*NAM*), *HD2B*, *PHAVOLUTA-like HD-ZIPIII*^34,35^, *PHLOEM INTERCALATED WITH XYLEM* (*PXY*^36^) (Table S2), however it shares more genes with the vascular cambium than with stem and root apical meristems or the flower meristem. Accordingly, several genes that were shown to be expressed in the inner layers of the skin where newly formed phellem cells are made, were also found to be expressed in the vascular cambium^37^. The phellogenic nature of the potato phellogen was further verified when its transcriptome was compared to the published transcriptome of bark (phellem and phellogen) from oak^21^, showing 68% of shared genes (Table S1, Column J). Hence, the overall data supported the similarity of the potato phellogen to the cambium of cork from woody species and to the vascular cambium.

The present transcriptome allowed the identification of cellular functions that play a role in the intensive proliferative activity of the phellogen. Functions that are related to cell division and differentiation, including histone modification and chromatin remodeling (Fig. 2), are dominant in meristems. Of the genes found to be highly expressed in the phellogen, the functional group related to ribosomal proteins (20%) was dominant (Fig. 2b). It was previously suggested that, in meristematic tissues, plants may use transcriptional control to synthesize extra ribosomes to increase translational efficiency^38^. The next largest functional group identified in our study was comprised of stress-associated genes, including heat-shock proteins that have been reported to play a role in regulation of cell cycle^39^ and in the dedifferentiation of meristems^40^. A model was suggested in which meristematic activity and stress responses share common mechanism. Stress-related responses promote genomic reprogramming to activate genes that are required for plant survival; similarly reprogramming of somatic cells (e.g., potato tuber hypodermis) is necessary to acquire an embryonic state (e.g., phellogen)^40^. Moreover, stress-related thioredoxin, catalase, glutathione-S-transferase and peroxidase (Fig. S1) were also shown to be involved in redox control of cell proliferation^39^. It has been demonstrated that homeostasis of reactive oxygen species (ROS) is required for cytokinesis, as ROS imbalance disrupt the building of the cell plate between daughter cells^41^.

High levels of vesicle trafficking and cell-wall synthesis are also seen in the phellogen (Fig. 2b). The deposition of a new cell wall between two daughter cells requires the formation of a cell plate whose formation is controlled by a cytoskeletal array known as the phragmoplast^42^. Cell-plate assembly requires the coordinated movement of cargo vesicles^43^ that transport cell-wall polysaccharides such as pectin and hemicellulose (synthesized in the Golgi), as well as cellulose, extensins and callose for mechanical stabilization of the newly formed cell wall. The vesicles are guided to the phragmoplast filaments by phragmoplast microtubule-binding myosin. The phellogen transcriptome includes 145 genes that are putatively related to cell-plate assembly (including genes that code for actin and myosin, as well as different aspects of vesicle trafficking, the cytoskeleton and microtubule organization). Some of these genes that are expressed at high levels in the phellogen transcriptome are considered markers of cytokinesis structure^42^. For example, but not limited to *ADP-RIBOSYLATION FACTOR 2* (Sotub01g008360), *ACT7* (Sotub03g020330), *VACUOLAR PROTEIN SORTING-ASSOCIATED PROTEIN 27* (Sotub04g025000), *RHD3* (Sotub03g035360), *MYOSIN* (Sotub03g013740); *RanGAP* (Sotub01g023990), *POK* (Sotub02g037270), *KATANIN* (Sotub09g022140), *EXOCYST* (Sotub04g023440) and *CALLOSE* (Sotub01g027340). The overall data point at the proliferative activity of the potato phellogen as its major characteristic.

### Identification of genes related to phellogen activation (skin initiation) and phellogen inactivation (skin-set)

Candidate genes for phellogen activity were selected based on their putative function and level of expression in the transcriptome, and their expression in defined developmental stages of the periderm (Fig. 3). These analyses allowed the identification of genes with differential and high expression levels during phellogen initiation and the early stages of skin formation, as compared to genes with differential and high expression following cessation of phellogen activity and the induction of skin maturation and skin-set processes (Fig. 4). Moreover, this unique differential expression was confirmed in the native periderm of two potato cultivars, Desirée and Rosanna (Figs 4 and S3), and in the wound-periderm model system (Fig. 5). The similar expression profiles of the phellogen-related genes of both Desirée and Rosanna suggest that the skin blemishes that are characteristic of the latter may be due to the low quality of phellem cell-wall composition—as was shown for trees^19,20^— rather than any interruption in phellogen cambial activity.

The specific phellogen activation/skin initiation-related genes included the histones *H2B*, *H3* and *H4*, the inducer of pro-cambial activity *VAS*, and the lignin/suberin-related *CCoAOMT1*, *CFT* and *GPAT3* – their main functions involve chromatin remodeling and cell-wall synthesis processes that are required for the establishment of the dedifferentiation state. As mentioned before, the initiation of potato phellogen requires the transition of somatic cells (hypodermis or tuber parenchyma cells) into pluripotent stem cells (phellogen) that can produce different types of progeny (i.e., phellem, phelloderm). During the somatic-to-meristematic transition, cells have to dedifferentiate, activate their cell-division cycle and reorganize their physiology, metabolism and gene-expression patterns^44^. Chromatin remodeling is an essential part of the coordinated reorganization of this cellular state. The basic structural unit of chromatin, the nucleosome, is made up of DNA wrapped around a histone octamer containing two copies of each of the four core histone proteins, H2A, H2B, H3, and H4 (reviewed in Kornberg and Lorch, 1999^45^). Thus, chromatin remodeling during dedifferentiation is carried out by histone proteins. For example, the H3 protein is incorporated during the S-phase and maintained at high levels in rapidly dividing cells, but is removed from cells undergoing their last cell cycle before exit to differentiation^46^. The histones H2B, H3 and H4, which are related to phellogen initiation activity (Fig. 4), form a putative protein interaction network that also includes other histone proteins from the transcriptome (Fig. S5, Table S3). In addition to histones, several markers of switch in cell fate for the somatic-to-meristematic transition can be found in the phellogen transcriptome. These include members of *AGAMOUS* transcription factor that is associated with maintaining the juvenile state of a tissue, the *SOMATIC EMBRYOGENESIS RECEPTOR KINASE* (*SERK*) capable of inducing embryogenic development in somatic cells, and the *WUSCHEL* (*WUS*) that putatively maintains the pluripotent state of the phellogen (Table S1 ^44^).

Another selected gene for phellogen-initiation activity, *VAS*, was reported to be involved in the initiation of pro-cambium from pericycle cells in Arabidopsis and the promotion of cell-division activity^47^. VAS protein forms an association network with phellogen proteins whose activities are related to chromatin regulation and cell-wall synthesis (Table 1).

The synthesis of the new cell wall following cytokinesis has to be integrated into the events of the cell cycle^44^. In accordance with this fact, three additional selected genes that were associated with phellogen initiation are putatively related to cell-wall biosynthesis. CCoAOMT1 and its associated proteins are involved in lignification of vascular tissue (Fig. 6, Table 1)^48^. The CFT with a GDSL lipase motif may be involved in suberin biosynthesis, and GPAT3, an ortholog of the Arabidopsis GPAT4, is putatively involved in the biosynthesis of glycerol and α,ω-dicarboxylic acids, which are components of the suberin polymer^49^. GPAT3-interacting proteins from the phellogen transcriptome are involved in glycerolipid metabolism as well (Fig. 6, Table 1).

Hence, results indicate that genes isolated for phellogen initiation stage involve in cytokinesis and chromatin reprograming for the establishment of a dedifferentiation state.

The phellogen inactivation/skin maturation (skin-set)-related genes included *HGF3/HUA2*, *PHDZnP/HAT3*, *ACT7*, *POD*, *APK1/AtATH8*, *BTH4/MED15* and *PED3/CTS* (Fig. 4), with a putative function in aging meristem. *HGF3/HUA2* acts together with *AGAMOUS* in specification of organ identity in meristem^50^. *PHDZnP/HAT3* controls meristem activity and organ polarity in an already established meristem, through changes in auxin distribution and response^51^. Its associated proteins from the phellogen transcriptome are REF6 and ELF6, which regulate brassinosteroid target gene expression through histone modification; many of these genes encode cell wall-modifying enzymes implicated in cell elongation, such as XYLOGLUCAN ENDOTRANSGLUCOSYLASES/HYDROLASES (XTHS), EXPANSINS and PECTATE LYASES^52,53^, which can also be found in the phellogen transcriptome (Table S1). A third gene, *MRG1*, a histone acetylation gene involved in photoperiodic regulation of flowering time^54^, was also identified; its role in the phellogen has yet to be clarified.

As to functions that are related to the cell wall of aging phellogen, ACT7 and the proteins with which it interacts may be part of the phragmoplast, providing the cytoskeletal array for the establishment of a new cell wall during cell division and directing cargo vesicles to the cell plate^42,43,55^. APK1/AtATH8 and PED3/CTS and the associated proteins from the phellogen transcriptome (LACS6 and PEX) are involved in transport and beta-oxidation of fatty acids^56^, and may play a role in suberization. BTH4/MED15 regulates glycolysis-related and fatty acid biosynthetic genes during embryogenesis^57^. POD may be involved with lignification and suberization and its associated proteins involved in lignin biosynthesis (Fig. S5, Table S3). Another member of the *POD* family (Sotub02g027930) that can be found in the phellogen transcriptome is the *POD 72-like* (Sotub02g027920) gene, which has been shown to be expressed in the tuber skin^37^. The above-described functions of cell wall synthesis and modification following phellogen inactivation are in agreement with histological and immunocytological data that revealed a thickening of phellogen radial walls that accompanied periderm maturation and increased resistance to skinning injuries^2,3,58^.

In conclusion, isolation of potato phellogen cells allowed the identification of cellular processes that characterize cork cambial activity, particularly those related to cell division, chromatin remodeling, stress-related activities, enhanced ribosomal activity and vesicle trafficking, and cell-plate synthesis. Similar activities could be identified in cork from trees, indicating common characteristics of the phellogen *in planta*. Moreover, expression analysis of selected genes pointed at the cellular processes that govern phellogen activation to form the periderm/cork, as well as phellogen inactivation to allow periderm/cork maturation. Phellogen activation involves cytokinesis and gene reprograming for the establishment of a dedifferentiation state; whereas maturing phellogen is characterized by activities that direct organ identity in the meristem and cell-wall modifications. The identification of phellogen-related processes and the respective genes provides, for the first time, information about the cellular and molecular functions related to cork cambium activity.

## Materials and Methods

### Plant material

Potato (*Solanum tuberosum* L.) cultivars Desirée and Rosanna were grown from tubers in 20-L containers in a greenhouse under natural winter conditions (December–January, average temperature range of 10–18 °C). The experiments were conducted with cv. Desirée unless stated otherwise. Tubers were harvested at three time-points: 3 weeks after sprout emergence (WAE), when phellogen cells are initiated; 8 WAE, when phellogen cells are actively dividing to form the immature skin; and at 12 WAE, when the periderm has matured following skin-set. Peels of tuber periderm (about 200 μm thick) were collected using a pedicure shaver to minimize tuber-flesh contamination. Tuber flesh was collected separately. Tissue samples were snap-frozen in liquid nitrogen and stored at −80 °C.

To isolate wound periderm, at 8 WAE potato tubers (cv. Desirée) were cut and the slices were kept under dark and humid conditions to allow healing^59^. The development of wound periderm was monitored every 24 h using UV and light microscopes and the forming wound periderm was collected from Day 1 through Day 8 after wounding. Collected tissue was snap-frozen in liquid nitrogen and stored at −80 °C.

Microtubers were induced in tissue culture according to a standard protocol^60^. Single-node stem cuttings were placed on a petri dish containing Nitsch medium (pH 5.8; N0224.0050, Duchefa Biochemie, Haarlem, The Netherlands) that included 8% (w/v) sucrose, 5 mg L^−1^ kinetin (Sigma), 2 mg L^−1^ ancimidol (Sigma) and 0.8% (w/v) agar (Sigma). The cuttings were incubated in the dark at 24 °C and microtuber initiation could be observed after 7 days.

### Reporter constructs for phellogen activity and GUS assay

Beta-glucuronidase (GUS) reporter constructs containing the promoter of the cell-division gene, *MITOTIC CYCLIN B1;1 (CycB1;1)*^23^, and the *ARABIDOPSIS RESPONSE REGULATOR 5* (*ARR5)*^24^, a cytokinin-responsive gene, were used to transform potato plants as described in Joshi *et al*., 2016^61^. To study the expression of the constructs in tuber tissues, cultures of *in vitro*-induced microtubers were prepared and free-hand sections of 8- to 10-day-old microtubers were subjected to GUS (#R0851, Thermo Scientific, Surrey, UK) staining based on Jefferson *et al*., 1987^62^.

### Microscopic studies

Tissue samples were fixed in FAA (50% ethanol, 5% acetic acid and 3.7% formaldehyde, v/v), dehydrated in an ethanol/xylene series and embedded in paraplast (Paraplast Plus, McCormick Scientific, St. Louis, MO), according to standard methods^63^. Tissue sections (15–20 µm) were stained with Safranin-O/Fast green (Sigma) for examination of tissue morphology. Sections were observed under a light microscope (Leica DMLB, Wetzlar, Germany) and images were displayed on a monitor through a CCD camera (Leica DC2000) using the Leica IM1000 program. The same samples were viewed under UV light to detect autofluorescence of suberized cell walls in the skin. The Leica DMLB microscope was configured for epifluorescent illumination using an HBO103W/2 mercury lamp, excitation filter BP 340–380, chromatic beam-splitter FT 400 and barrier filter LP 425.

For the isolation of potato phellogen cells by LCM it was required to confront the following challenges: (a) the phellogen is one cell layer in the periphery of the tuber, (b) phellogen cell morphology is similar to the phellem cell above it, and (c) no staining procedure or UV illumination can be used to identify it during the LCM procedure. Nevertheless, at a certain early stage of phellogen activity the cells exhibit puffy morphology that distinguish them from other periderm cells. To identify this exact stage it was necessary to screen microscopically high number of tubers with immature skin. To this end, tubers of greenhouse-grown potatoes were collected at early stages of tuber development, starting from the tuberization stage at around 3–4 WAE and extending up to 8 WAE, when skin-formation activity is at its highest level^64^. The samples were embedded in paraplast as described above, except using the Farmer’s fixative (ethanol and acetic acid in 3:1 ratio) instead of FAA. Sections (15 µm thick) of all tissue samples were observed under a light microscope to select the developmental stage at which the actively dividing phellogen cells could be clearly identified by their puffy morphology, as demonstrated in Fig. 1c. Tissue blocks from 6 WAE were selected based on best visualization of the phellogen, sectioned and placed on a membrane slide (1.0 PEN, cat no: 415190-90410-000, Carl Zeiss microscopy GmbH, Gottingen, Germany). Phellogen cells (~4150 cells) were collected using the LCM (PALM MicroBeam ZEISS Microscopy) at a service unit of Ben-Gurion University, Israel. Isolated cells were immediately resuspended in 350 µl of RLM buffer [4 M guanidine isothiocyanate, 0.2 M sodium acetate, 25 mM EDTA, 2.5% (w/v) PVP-40], frozen in liquid nitrogen and stored at −80 °C.

### RNA extraction and amplification

Extraction of total RNA from tuber skin and flesh was done according to Ginzberg *et al*., 2009^64^. Extraction of RNA from LCM-isolated phellogen cells was done using the RNeasy Plus Micro Kit (cat no. 74034, Qiagen, Germany). The isolated RNA was amplified (three rounds) using the Message Amp^TM^ II aRNA Amplification Kit (cat. no. AM1751, Ambion™, Thermo Fisher Scientific, USA). A total amount of 0.7 µg was sent for Illumina sequencing at the Technion Genome Center (Haifa, Israel).

### Transcriptome data analysis

The transcriptome reads were aligned to the potato genome sequence (PGSC_DM_Version 3.4) available at Sol Genomics Network site (https://solgenomics.net/) using the Tophat2 program. Cleaning, trimming and quality filtering of the data were done using the FastX tool kit (http://hannonlab.cshl.edu/fastx_toolkit/) with the help of bioinformatics services available at ARO. Reads smaller than 100 bp that were not mapped to the genome and could not be verified as “true” genes, were removed from the data. Annotation was performed by BLAST alignment to the potato, tomato and Arabidopsis databases. Blast2go version 4.0 (https://www.blast2go.com/) was used for Gene Ontology (GO) assignments. Genes that were highly expressed in the phellogen and genes that were of interest based on their general function were further annotated by screening the literature for better categorization. The MapMan software (MapMan 3.6.0RC1; https://mapman.gabipd.org/) was used to sort the transcriptome into functional groups based on the TAIR IDs of the potato orthologs. The transcriptome FPKM (fragments per kilobase million) values with restricted scale to ± 100 were used.

The STRING protein-protein interaction network (version 10.5; https://string-db.org/)^64^ was used to analyze putative protein-association networks within the phellogen transcriptome data. For this, Arabidopsis orthologs of selected potato phellogen genes were entered into the STRING search, limiting the minimum required interaction score of 0.4. Protein members of an interactome were listed and used to back-search the phellogen transcriptome data based on their TAIR IDs. A putative function of the interactome with respect to phellogen activity was suggested.

### Gene-expression studies using quantitative real-time PCR (qPCR)

cDNA was synthesized using EZ-First Strand cDNA Synthesis Kit for qRT-PCR (Biological Industries, Beit Haemek, Israel). Taq polymerase (Super-Therm 500 u, cat. no. JMR-801 PCR, JMR Holdings, London, UK) was used for semi-quantitative PCR and ABsolute™ Blue QPCR SYBR^®^ Green ROX Mix (Thermo Scientific, Surrey, UK) was used for qPCR, according to the manufacturer’s protocol, with specific primers (Table S4). Primers were designed based on the phellogen RNA-seq data. In some cases, primers were designed based on their respective gene sequences in the potato genome available through the Sol Genomics Network. Each qPCR was performed with three biological replicates, each with three technical replicates. Values in each sample were normalized to the levels of α-chain of the nascent polypeptide-associated complex (α-*NAC*, Sotub10g027110^64^, *ACTIN* (*ACT*, Sotub10g022240) and *UBIQUITIN EXTENSION PROTEIN* (*UBQ*, Sotub12g030900). All resulted with similar expression pattern; the normalization versus the α-*NAC* is shown. Statically significant differences between means were identified using Student’s *t*-test (JMP software, http://www.jmp.com). Significance was determined at *P* < 0.05.

### Isolation of phellogen-selected genes

The full coding regions of selected genes were isolated using Ex-Taq polymerase with proofreading activity (TaKaRa Clontech, Kusatsu, Shiga, Japan), gene-specific primers (CDS primers, Table S1) and periderm peel from 3- and 12-WAE-old tubers. cDNA fragments were cloned into the pENTR^TM^/D-TOPO (ref. no. 45-0218, cat no. K2400-20, Invitrogen, USA) vector according to the manufacturer’s protocol and transformed into One Shot TOP10 Chemically Competent *E*. *coli* cells (cat. no. C4040-03, Invitrogen, USA). Inserts were verified by sequencing. Sequence data can be found in the GenBank data libraries under accession numbers *VAS*, MH669350; *CCoAOMT1*, MH669351; *CFT*, MH669352; *PHDZnP/HAT3*, MH669353; *MTN*, MH669354; *OSP*, MH669355; *BTH4/MED15*, MH669356; *H2B*, MH669357; *H3*, MH669358; *H4*, MH669359; *ACT7*, MH669360; *GPAT3*, MH669361; *POD*, MH669362; *PED3/CTS*, MH669363.

## Supplementary information

Supplementary Figures

Supplementary Table

Supplementary Tables
