# Supplementary material for: The transcriptome of potato tuber phellogen reveals cellular functions of cork cambium and genes involved in periderm formation and maturation
Source: Sci Rep. 2019 Jul 15;9:10216. doi: 10.1038/s41598-019-46681-z (PMC6629697; doi:10.1038/s41598-019-46681-z)
Supplement: Supplementary file 1 — Supplementary Figures [file 41598_2019_46681_MOESM1_ESM.pdf]

Supplementary information

The transcriptome of potato tuber phellogen reveals cellular functions of cork cambium and genes involved in periderm formation and maturation

Vijaya K. R. Vulavala[1,2], Edna Fogelman[1], Adi Faigenboim[1], Oded Shoseyov[2], Idit Ginzberg[1,*]

[1]Institute of Plant Sciences, Agricultural Research Organization, Volcani Center, 68 HaMacabim Road, P. O. Box 15159, Rishon LeZion, 7505101, Israel

[2]The Robert H. Smith Institute of Plant Sciences and Genetics in Agriculture, Faculty of Agriculture, Hebrew University of Jerusalem, Rehovot 7610001, Israel

* Corresponding author, E-mail: iditgin@volcani.agri.gov.il

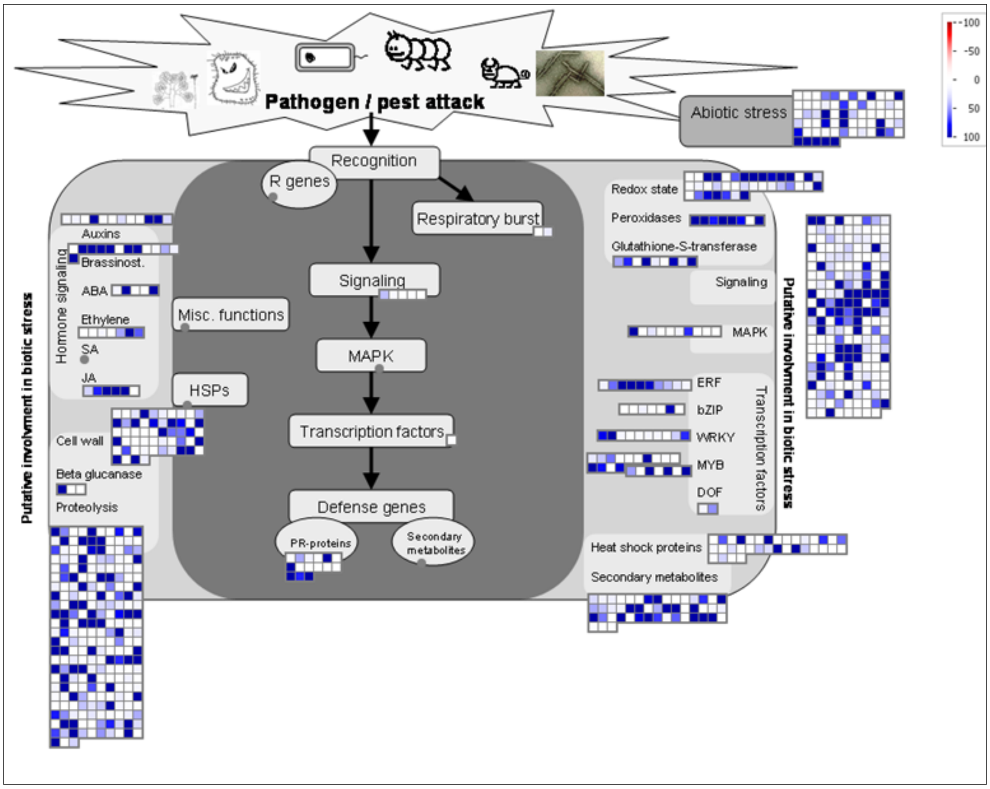

Figure S1. An overview of phellogen-related stress functions analyzed by the MapMan application (version 3.6.0RC1; https://mapman.gabipd.org/) using Arabidopsis orthologs of the phellogen transcriptome. The number of squares presented for each function indicates the number of transcripts that belong to that group. The gradient in color intensity represents the FPKM of each transcript (FPKM restricted to ±100).

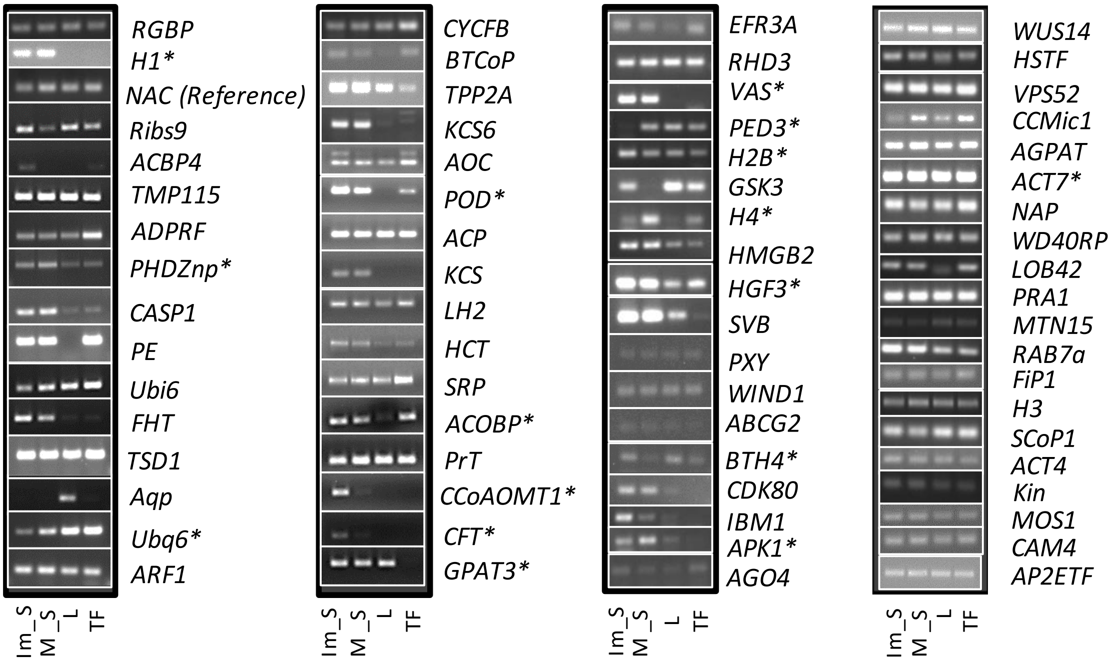

Figure S2a. Differential expression of selected genes from the phellogen transcriptome to verify periderm/skin specific expression. Expression profiles were examined in immature skin (Im_S) and mature skin (M_S), collected at 6 and 12 weeks after sprout emergence, and in leaf tissue (L) and tuber flesh (TF). Information about the selected genes is provided in Table S1, Column W. Genes with relatively high levels of expression in immature skin (labelled with an asterisk) were selected for further analysis by qPCR (see Fig. S2b, Fig. 4 and Fig. 5).

This figure was composed of several agarose gels. Expression signals for each gene were cut from gel pictures and rearranged in a form of a table as seen in this figure.

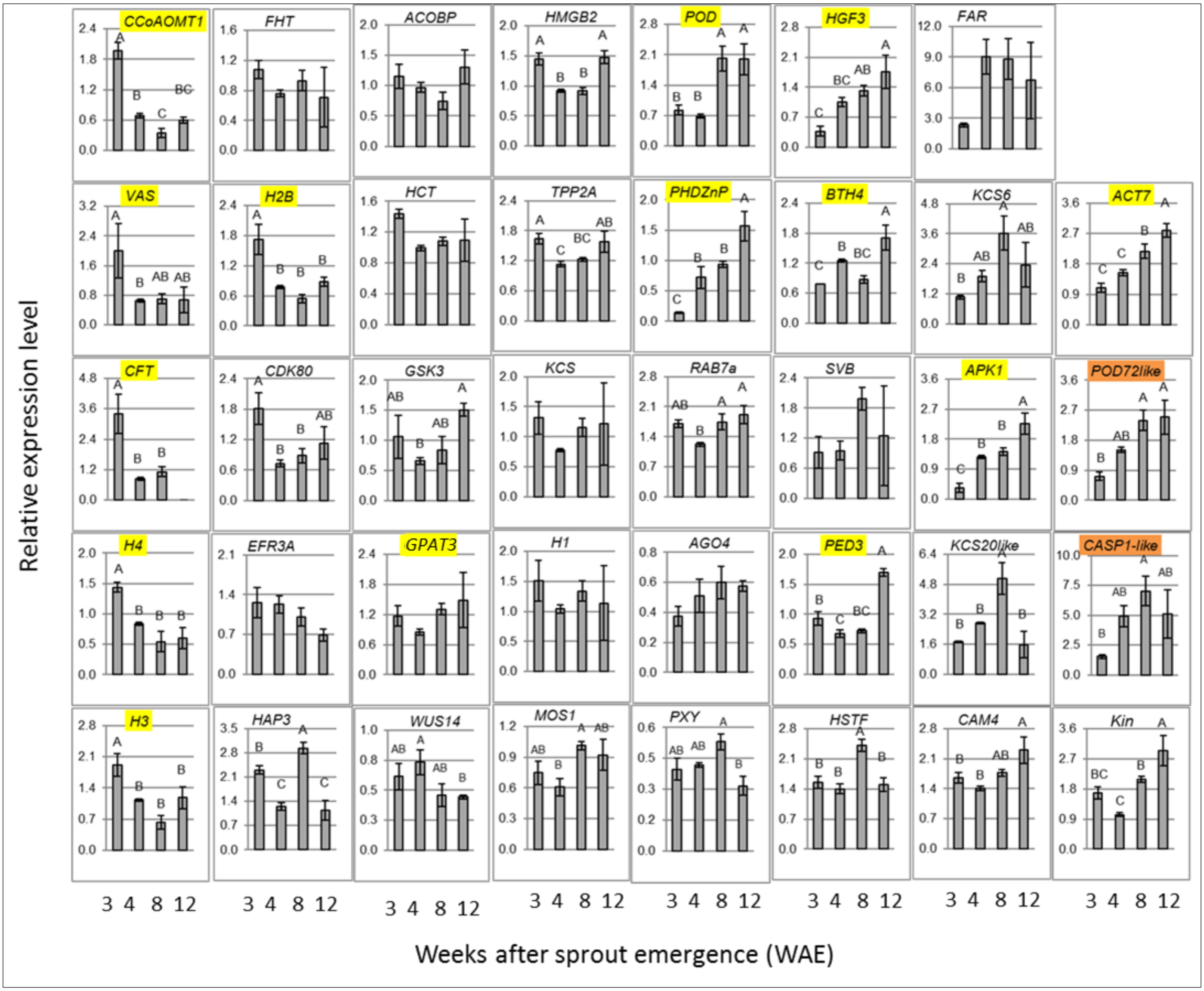

Figure S2b. Expression profiles of selected genes that were expressed at high levels in the skin, but were not expressed in the leaf tissue or tuber flesh, at four defined stages of periderm development. Periderm peels were collected: at phellogen/skin initial stage (3 WAE), at skin formation (4 WAE), from immature skin (8 WAE) and from mature skin following skin-set (12 WAE). Transcript levels were monitored by qPCR and values were normalized relative to the level of the reference gene *NAC*. Genes with differential and high expression following skin initiation and genes with differential and high expression following skin maturation were chosen for further analysis and are highlighted in yellow. Skin-related genes that were previously identified by us (Vulavala et al. (32)) were also found in the phellogen transcriptome (highlighted in orange), and were also highly expressed following skin maturation, as expected. Values represent an average of three biological replicates with SE bars. Statistically significant differences between means were identified using Student's *t*-test; different letters indicate significantly different values ($P < 0.05$).

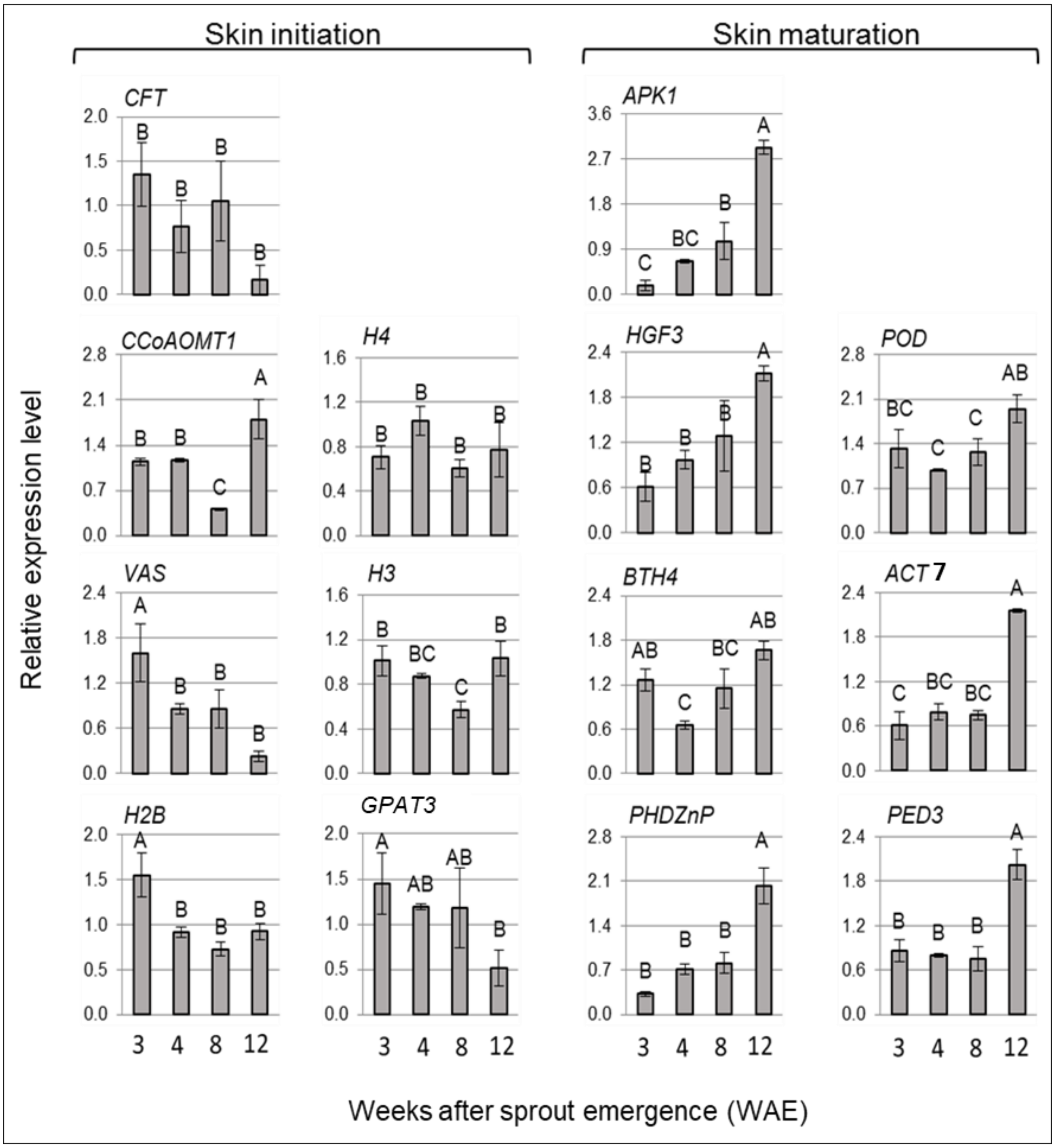

Figure S3. Expression profiles of phellogen-related genes at four stages of periderm development collected from cv. Rosanna, which is prone to skinning injuries. Periderm peels were collected: at phellogen/skin initial stage (3 WAE), at skin formation (4 WAE), from immature skin (8 WAE) and from mature skin following skin-set (12 WAE). (Skin anatomy at the respective stages is described in S2 Fig) Transcript levels were monitored by qPCR and expression levels were normalized relative to the level of the reference gene *NAC*. Genes that exhibited differential and high expression during skin initiation are presented on the left side of the figure and genes that are expressed mainly in the mature skin are presented on the right. Values represent an average of three biological replicates with SE bars. Statistically significant differences between means were identified using Student's *t*-test; different letters indicate significantly different values ($P < 0.05$).

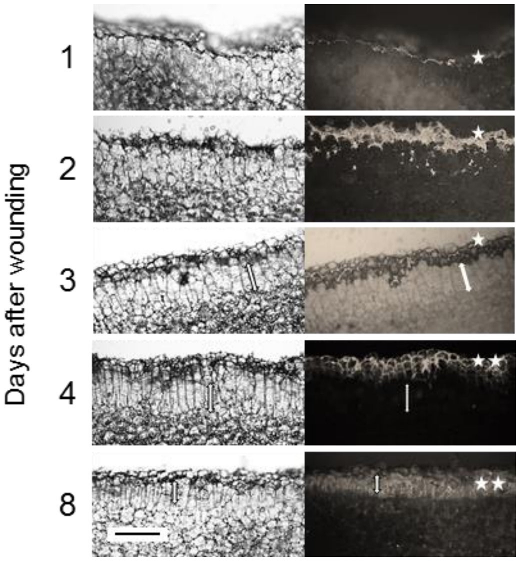

Figure S4. Developmental stages of potato wound periderm. Wound periderm develops at the surface of tuber slices kept in the dark under high humidity (5). Free-hand sections of the slices were made at 1-day intervals following the slicing, and viewed under a light microscope (left panel) and a UV microscope (right panel, black background), to examine tissue morphology and the autofluorescence of suberized cells, respectively.

Wounding induced the formation of a closing layer, in which cell walls of the exposed tuber parenchyma cells underwent lignification/suberization (Days 1–3, marked with a star). On Day 3, columns of new phellem cells could be clearly seen below the closing layer (marked with an arrow), indicating phellogen initiation around 1 to 2 days after wounding. From Day 4 on, the newly formed phellem underwent suberization, from the outside inward (marked with two stars). On Day 8, the phellem cells were suberized and their layers were compressed, indicating the maturation of the wound periderm. Bar: 500 µm.

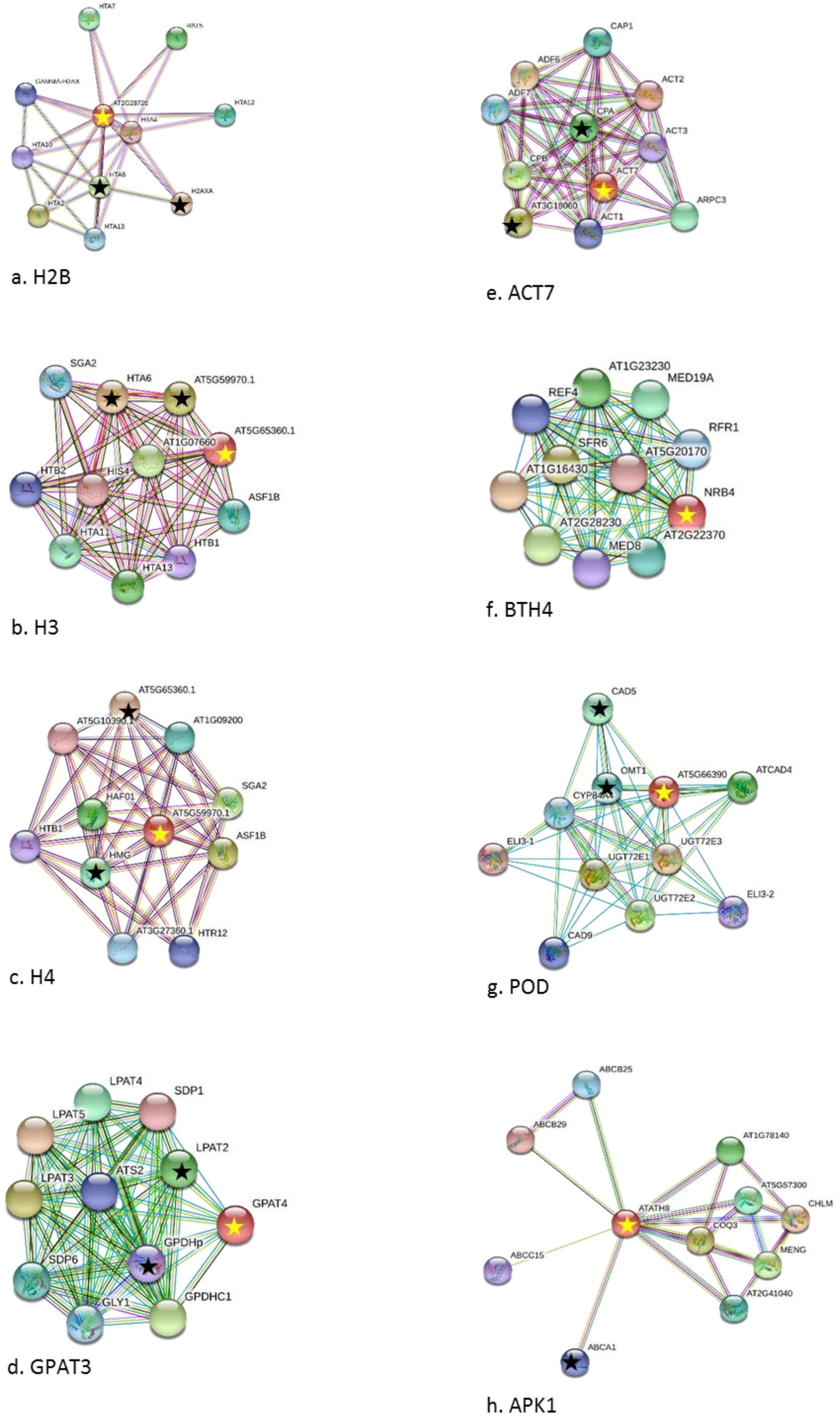

HTA7
GAMMA-H2AX
AT2G28720
HTA4
HTA12
HTA10
HTA6
H2AXA
HTA2
HTA13
a. H2B
SGA2
HTA6
AT5G59970.1
AT5G65360.1
AT1G07660
HTB2
HIS4
ASF1B
HTA11
HTB1
HTA13
b. H3
AT5G65360.1
AT5G10390.1
AT1G09200
SGA2
HAF01
AT5G59970.1
HTB1
ASF1B
HMG
AT3G27360.1
HTR12
c. H4
LPAT4
SDP1
LPAT5
LPAT2
LPAT3
ATS2
GPAT4
GPDHp
SDP6
GLY1
GPDHC1
d. GPAT3
CAP1
ADF6
ACT2
ADF7
CPA
ACT3
CPB
ACT7
AT3G18060
ACT1
ARPC3
e. ACT7
AT1G23230
MED19A
REF4
RFR1
SFR6
AT5G20170
AT1G16430
NRB4
AT2G28230
AT2G22370
MED8
f. BTH4
CAD5
OMT1
AT5G66390
ATCAD4
CYP84A4
ELI3-1
UGT72E3
UGT72E1
UGT72E2
ELI3-2
CAD9
g. POD
ABCB25
ABCB29
AT1G78140
AT5G57300
ATATH8
CHLM
COQ3
ABCC15
MENG
AT2G41040
ABCA1
h. APK1

Figure S5

Figure S5. STRING: functional protein association networks (https://string-db.org/) of selected phellogen-related genes. Arabidopsis orthologs of the potato genes were identified and their TAIR IDs were entered into the STRING search engine. For each gene cluster, the query phellogen gene is marked with a yellow star and putative associated proteins that were also found in the potato phellogen transcriptome are marked with a black star. Data on the members of the interactomes is given in S2 Table, Column Y, and S4 Table. The interactomes of histone proteins (a) H2B, (b) H3 and (c) H4, included the phellogen-related common proteins H2A6 and H2AXa. All involved in chromatin remodeling, which is essential in the coordinated reorganization of somatic cell state to dedifferentiation during the meristematic transition of phellogen cells. (d) The interactome of GPAT3 included the phellogen-related GPDHp (GLYCEROL-3-PHOSPHATE DEHYDROGENASE) and LPAT2 (LYSOPHOSPHATIDYL ACYLTRANSFERASE 2), which are putatively involved in cell-wall suberin biosynthesis. (e) The interactome of ACT7 (ACTIN 7) included the phellogen-related CPA (CAPPING PROTEIN A) and TRANSDUCIN/WD40 DOMAIN-CONTAINING PROTEIN, which provide the cytoskeletal array for the establishment of a new cell wall during cell division and direct cargo vesicles to the cell plate in phellogen. (f) The interactome of BTH4/MED15 included proteins involved in cell-wall biosynthesis, but none were found in phellogen transcriptome. (g) The interactome of POD included phellogen-related CAD5 (CINNAMOYL ALCOHOL DEHYDROGENASE 5) and OMT1 (*O*-METHYL TRANSFERASE 1), which are involved in cell-wall lignin biosynthesis. (h) The interactome of APK1 included the phellogen-related ATP-BINDING CASSETTE 1, which is involved in membrane transport of fatty acids and may be involved in cell-wall suberization.

.
