# Supplementary material for: The transcriptome of potato tuber phellogen reveals cellular functions of cork cambium and genes involved in periderm formation and maturation
Source: Sci Rep. 2019 Jul 15;9:10216. doi: 10.1038/s41598-019-46681-z (PMC6629697; doi:10.1038/s41598-019-46681-z)
Supplement: Supplementary file 3 — Supplementary Tables [file 41598_2019_46681_MOESM3_ESM.pdf]

Supplementary information

The transcriptome of potato tuber phellogen reveals cellular functions of cork cambium and genes involved in periderm formation and maturation

Vijaya K. R. Vulavala[1,2], Edna Fogelman[1], Adi Faigenboim[1], Oded Shoseyov[2], Idit Ginzberg[1,*]

[1]Institute of Plant Sciences, Agricultural Research Organization, Volcani Center, 68 HaMacabim Road, P. O. Box 15159, Rishon LeZion, 7505101, Israel

[2]The Robert H. Smith Institute of Plant Sciences and Genetics in Agriculture, Faculty of Agriculture, Hebrew University of Jerusalem, Rehovot 7610001, Israel

* Corresponding author, E-mail: iditgin@volcani.agri.gov.il

Table S2 List of meristem-related genes found in the phellogen transcriptome

Gene name	Sol genomics ID	TAIR ID	Reference
**Cambium**			
ACA8	Sotub07g009770.1.1	AT5G57110.2	[1]
AGO4	Sotub01g009760.1.1	AT2G27040.2	[1]
APH3	Sotub01g022470.1.1	AT3G21510.1	[1]
Aquaporin	Sotub03g036560.1.1	AT3G16260.1	[2]
Aquaporin	Sotub01g014220.1.1	AT3G56950.2	[2]
Aquaporin	Sotub06g009110.1.1	AT4G00430.1	[2]
Aquaporin	Sotub01g032080.1.1	AT4G23400.1	[2]
Aquaporin	Sotub01g049070.1.1	AT4G35100.2	[2]
ARF3	Sotub02g020520.1.1	AT2G33860.1	[3]
ARF4	Sotub01g036930.1.1	AT1G54990.1	[3]
ATHB-15 [HD-ZipIII]	Sotub03g036370.1.1	AT1G79840.2	[3]
ATHB9	Sotub01g030790.1.1	AT4G00730.1	[3]
CDK	Sotub01g029100.1.1	AT2G44740.1	[2]
CDK	Sotub01g029950.1.1	AT3G60550.1	[2]
CDK inhibitor	Sotub12g030040.1.1	AT2G32710.2	[2]
CDK inhibitor	Sotub09g017820.1.1	AT5G48820.1	[2]
Cyclin	Sotub01g025990.1.1	AT5G21080.1	[2]
Cyclin A	Sotub03g029000.1.1	AT2G18540.1	[2]
Cyclin B	Sotub02g026170.1.1	AT1G20610.1	[2]
Cyclin D3	Sotub02g031650.1.1	AT4G34090.1	[4]
Cyclin D3:1	Sotub02g031450.1.1	AT5G67260.1	[4]
Cyclin D3:2	Sotub01g044850.1.1	AT5G67260.1	[4]
DNA methyltransferase-associated protein (DMAP1)	Sotub01g023480.1.1	AT2G47210.1	[5]
DRM-type DNA-methyltransferase	Sotub02g010360.1.1	AT5G14620.1	[5]
Expansin	Sotub02g034760.1.1	AT1G26770.2	[2]
Expansin	Sotub02g024320.1.1	AT3G03220.1	[2]
Expansin	Sotub01g049420.1.1	AT3G45970.1	[2]
GSK3	Sotub01g040370.1.1	AT1G09840.6	[6]
GSK3	Sotub01g030780.1.1	AT4G00720.1	[6]
GSK3	Sotub02g018890.1.1	AT4G18710.1	[6]
GSK3	Sotub02g036240.1.1	AT5G14640.1	[6]
H2B	Sotub03g016600.1.1	AT2G28720.1	[2]
H2B	Sotub02g018820.1.1	AT4G18740.1	[2]
H4	Sotub11g029670.1.1	AT5G59970.1	[2]
PXY	Sotub02g030330.1.1	AT5G65700.2	[1]
REVERSION-TO-ETHYLENE SENSITIVITY1 (RTE1)	Sotub02g010060.1.1	AT3G51040.3	[1]
RUL1	Sotub03g018340.1.1	AT5G58300.2	[1]

SCRAMBLED (SCM)	Sotub02g021640.1.1	AT2G20850.1	[1]
VASCULAR TISSUE SIZE (VAS)	Sotub01g040060.1.1	AT5G13900.1	[7]
VASCULAR TISSUE SIZE (VAS)	Sotub01g042060.1.1	AT5G64080.1	[7]
XET/XTH	Sotub03g008310.1.1	AT1G11545.1	[2]
XET/XTH	Sotub01g028070.1.1	AT1G32170.1	[2]
XET/XTH	Sotub12g019260.1.1	AT4G25810.1	[2]
XET/XTH	Sotub12g019250.1.1	AT4G25810.1	[2]
XET/XTH	Sotub01g037390.1.1	AT5G13870.1	[2]
**Shoot apical meristem**			
HD2 (HD2B)	Sotub11g024650.1.1	AT3G44750.2	[8]
MRG 1	Sotub02g031380.1.1	AT4G37280.1	[9]
No Apical Meristem (NAM)	Sotub03g017140.1.1	AT3G49530.1	[10]
No Apical Meristem (NAM)	Sotub03g029340.1.1	AT5G61430.1	[10]
No Aplical Meristem (NAM)	Sotub04g009390.1.1	AT1G01720.1	[10]
PHAVOLUTA-like HD-AIPIII protein	Sotub02g016070.1.1	AT2G34710.1	[11]
RING1B	Sotub02g020780.1.1	AT5G44280.1	[12]
RINGLET2 (RTL2)	Sotub02g020390.1.1	AT1G28420.1	[13]
TOPLESS (TPL)	Sotub03g030200.1.1	AT3G15880.1	[14]
Topless-Related 2 (TPR2)	Sotub01g037850.1.1	AT5G27030.1	[15]
**Floral meristem**			
CONSTANS	Sotub01g025960.1.1	AT3G63500.2	[16]
CONSTANS	Sotub01g047820.1.1	AT4G38960.1	[16]
CONSTANS	Sotub02g022400.1.1	AT5G54470.1	[16]
CYCLING DOF FACTOR 2 (CDF2)	Sotub02g034790.1.1	AT5G39660.2	[17]
Jumonji	Sotub04g018130.1.1	AT5G04240.1	[18]
RED AND FAR-RED INSENSITIVE 2 (RF12)	Sotub01g037570.1.1	AT3G05545.1	[19]
SCM	Sotub02g021640.1.1	AT2G20850.1	[1]
SFH12	Sotub01g047280.1.1	AT2G21540.2	[20]
StJMJ16	Sotub04g009950.1.1	AT1G08620.2	[21]
TFL2	Sotub01g027600.1.1	AT5G17690.1	[22]
**Root meristem**			
ACAR FAMILY PROTEIN 4 (AtSCAR4)	Sotub02g019430.1.1	AT2G34150.2	[23]
CYTOCHROME C-2 (CYTC-2)	Sotub01g040200.1.1	AT4G10040.1	[24]
HAWAIIAN SKIRT (HS/HWS)	Sotub01g032770.1.1	AT3G61590.2	[25]
MPK6	Sotub05g022310.1.1	AT4G01370.1	[26]
RanGap 1	Sotub01g023990.1.1	AT5G19320.1	[26]
TONNEAU 1	Sotub04g009490.1.1	AT3G55000.1	[26]
TONNEAU 2/FASS	Sotub01g021510.1.1	AT5G18580.1	[26]

References

1 Agusti, J., Lichtenberger, R., Schwarz, M., Nehlin, L. & Greb, T. Characterization of transcriptome remodeling during cambium formation identifies *MOL1* and *RUL1* as opposing regulators of secondary growth. *PLOS Genet.* **7**, e1001312, doi:10.1371/journal.pgen.1001312 (2011).

2 Schrader, J. *et al.* A high-resolution transcript profile across the wood-forming meristem of poplar identifies potential regulators of cambial stem cell identity. *Plant Cell* **16**, 2278-2292 (2004).

3 Ramachandran, P., Carlsbecker, A. & Etchells, J. P. Class III HD-ZIPs govern vascular cell fate: an HD view on patterning and differentiation. *J Exp Bot* **68**, 55-69 (2017).

4 Collins, C., Maruthi, N. M. & Jahn, C. E. *CYCD3* D-type cyclins regulate cambial cell proliferation and secondary growth in Arabidopsis. *J. Exp. Bot.* **66**, 4595-4606 (2015).

5 Ramos, M., Rocheta, M., Carvalho, L., Graça, J. & Morais-Cecilio, L. Expression analysis of DNA METHYLTRANSFERASE and co-repressor genes in *Quercus suber* phellogen: an attempt to correlate with cork quality. *BMC Proc* **5**, P169, doi:10.1186/1753-6561-5-S7-P169 (2011).

6 Kondo, Y. *et al.* Plant GSK3 proteins regulate xylem cell differentiation downstream of TDIF–TDR signalling. *Nature Commun.* **5**, 3504 (2014).

7 Van Der Graaff, E., Hooykaas Paul, J. J. & Keller, B. Activation tagging of the two closely linked genes *LEP* and *VAS* independently affects vascular cell number. *Plant J.* **32**, 819-830 (2002).

8 Han, Z. *et al. AtHD2D* gene plays a role in plant growth, development, and response to abiotic stresses in *Arabidopsis thaliana*. *Front. Plant Sci.* **7**, doi:10.3389/fpls.2016.00310 (2016).

9 Bu, Z. *et al.* Regulation of Arabidopsis flowering by the histone mark readers *MRG1/2* via Interaction with *CONSTANS* to modulate *FT* expression. *PLOS Genet.* **10**, e1004617, doi:10.1371/journal.pgen.1004617 (2014).

10 Souer, E., van Houwelingen, A., Kloos, D., Mol, J. & Koes, R. The *NO APICAL MERISTEM* gene of petunia is required for pattern formation in embryos and flowers and is expressed at meristem and primordia boundaries. *Cell* **85**, 159-170 (1996).

11 Yamada, T., Sasaki, Y., Hashimoto, K., Nakajima, K. & Gasser, C. S. *CORONA, PHABULOSA* and *PHAVOLUTA* collaborate with *BELL1* to confine *WUSCHEL* expression to the nucellus in Arabidopsis ovules. *Development* **143**, 422-426 (2016).

12 Xu, L. & Shen, W.-H. Polycomb silencing of *KNOX* genes confines shoot stem cell niches in Arabidopsis. *Curr. Biol.* **18**, 1966-1971 (2008).

13 Li, G. *et al.* Imitation switch chromatin remodeling factors and their interacting RINGLET proteins act together in controlling the plant vegetative phase in Arabidopsis. *Plant J.* **72**, 261-270 (2012).

14 Espinosa-Ruiz, A. *et al. TOPLESS* mediates brassinosteroid control of shoot boundaries and root meristem development in Arabidopsis thaliana. *Development* **144**, 1619-1628 (2017).

15 Zermiani, M. *et al.* Identification of the Arabidopsis RAM/MOR signalling network: adding new regulatory players in plant stem cell maintenance and cell polarization. *Ann. Bot.* **116**, 69-89 (2015).

16 Blázquez, M. A. Illuminating flowers: *CONSTANS* induces *LEAFY* expression. *Bioessays* **19**, 277-279 (1997).

17 Fornara, F. *et al.* Arabidopsis *DOF* transcription factors act redundantly to reduce *CONSTANS* expression and are essential for a photoperiodic flowering response. *Dev. Cell* **17**, 75-86 (2009).

18 Heidari, B., Nemie-Feyissa, D., Kangasjärvi, S. & Lillo, C. Antagonistic regulation of flowering time through distinct regulatory subunits of protein phosphatase 2A. *PLOS ONE* **8**, e67987, doi:10.1371/journal.pone.0067987 (2013).

19 Chen, M. & Ni, M. RFI2, a RING-domain zinc finger protein, negatively regulates *CONSTANS* expression and photoperiodic flowering. *Plant J.* **46**, 823-833 (2006).

20 Mo, P. *et al.* Identification of two phosphatidylinositol/phosphatidylcholine transfer protein genes that are predominately transcribed in the flowers of *Arabidopsis thaliana*. *J. Plant Physiol.* **164**, 478-486 (2007).

21 Shi, Y. Histone lysine demethylases: emerging roles in development, physiology and disease. *Nat Rev Genet* **8**, 829-833 (2007).
22 Larsson, A. S., Landberg, K. & Meeks-Wagner, D. R. The *TERMINAL FLOWER2 (TFL2)* gene controls the reproductive transition and meristem identity in *Arabidopsis thaliana*. *Genetics* **149**, 597-605 (1998).
23 Parizot, B., Roberts, I., Raes, J., Beeckman, T. & Smet, I. D. In silico analyses of pericycle cell populations reinforce their relation with associated vasculature in Arabidopsis. *Philos. Trans. R. Soc. B. Biol. Sci.* **367**, 1479-1488 (2012).
24 Welchen, E. & Gonzalez, D. H. Differential expression of the ARBIDOPSIS CYTOCHROME C genes CYTC-1 and CYTC-2. Evidence for the involvement of TCP-domain protein-binding elements in anther- and meristem-specific expression of the CYTC-1 gene. *Plant Physiol.* **139**, 88-100 (2005).
25 Kim, E. S. *et al. HAWAIIAN SKIRT* regulates the quiescent center-independent meristem activity in Arabidopsis roots. *Physiol. Plant.* **157**, 221-233 (2016).
26 Sotta, N., Shantikumar, L., Sakamoto, T., Matsunaga, S. & Fujiwara, T. TPR5 is involved in directional cell division and is essential for the maintenance of meristem cell organization in *Arabidopsis thaliana*. *J. Exp. Bot.* **67**, 2401-2411 (2016).

Table S3 Functional protein association networks within the phellogen transcriptome. Analysis was performed using STRING program (https://string-db.org/) based on Arabidopsis orthologs of phellogen-selected genes. Table lists interactomes based on Fig S5

Phellogen query	Query annotation	Members of interactome	TAIR ID	Interacted protein function
H2B (AT2G28720.1)	Histone H2B; core component of nucleosome	H2AXA (histone H2AXa)	AT1G08880.1	Chromatin remodeling
		HTA6 (Histone H2A 6)	AT5G59870.1	Chromatin remodeling
H3 (AT5G65360.1)	Histone H3; core component of nucleosome	Histone H4	AT5G59970.1	Chromatin remodeling
		HTA6	AT5G59870.1	Chromatin remodeling
H4 (AT5G59970.1)	Histone H4; core component of nucleosome	Histone H3	AT5G65360.1	Chromatin remodeling
		HMG (High mobility group)	AT3G28730.1	Chromatin remodeling
GPAT3/GPAT4 (AT1G01610.1)	Glycerol-3-phosphate acyltransferase; cutin biosynthesis	GPDHp (Glycerol-3-phosphate dehydrogenase)	AT5G40610.1	Glycerolipid metabolism
		LPAT2 (Lysophosphatidyl acyltransferase 2)	AT3G57650.1	Glycerolipid metabolism
ACT7 (AT5G09810.1)	Actin 7; cytoskeleton array	transducin/WD40 domain-containing protein	AT3G18060.1	Actin filaments elongation during cell divisions
		CPA (Capping protein A)	AT3G05520.2	Actin filaments elongation during cell divisions
BTH4/MED15 (AT1G15780.1)	Uncharacterized protein	NA	NA	NA
POD (AT5G66390.1)	Peroxidase 72; lignin/suberin biosynthesis	CAD5 (Cinnamyl alcohol dehydrogenase 5)	AT4G34230.1	Lignin biosynthesis
		OMT1 (*O*-Methyltransferase 1)	AT5G54160.1	Lignin biosynthesis
APK1/ATH8 (AT2G39190)	Transport and beta-oxidation of fatty acids	ABCA1 (ATP-binding cassette A1)	AT2G41700.1	Transport and beta-oxidation of fatty acids

Table S4 Primers used in this study

Gene Name	Sol genomics ID	NCBI ID	TAIR ID	Forward primer	Reverse primer	Amplicon size (bp)
**CDS**						
ACT7	Sotub03g020330	XM_006355076	AT5G09810	CACCGATTAAAAATGGCCGACGG	CAAACAACCTAAAAGAGACAC	1420
BTH4	Sotub04g009440	XM_006359266	AT1G15780	CACCGAGATGAATGGGAATAATTG	CACTAGGAAACTTGACAATC	832
CCoAOMT1	Sotub02g031720	XM_006339817	AT4G34050	CACCGCAGTTATCAATGACAAGC	CTTGAACTTAATAGGTACATG	951
CFT	Sotub01g036860	XM_006367783	AT1G28590	CACCAATGGCGTTGACAGTTAGC	CGATGATTTATGTTGTTATTTG	1303
GPAT3	Sotub01g032090	XM_006339523	AT1G01610	CACCAAAATGTCACTTCCAAAATCC	GACCTTAACAAGCCCGAGGG	1823
H2B	Sotub03g016600	XM_006364383	AT2G28720	CACCGCAAACAATGGCACCAAAGG	CATTCAAGGAATAGCTATG	583
H3	Sotub10g009520	XM_006360980	AT5G65360	CACCCCCAAATGGCTCGTACCAAG	GAATCGAGTAAATTCTGAATC	517
H4	Sotub11g029670	XM_006363280	AT5G59970	CACCCAAAAATGTCTGGCCGTGG	CAGAAACATAACCAATTACATC	390
MTN	Sotub06g010040	XM_006361145	AT5G02380	CACCTTATAATGTCTGGTTGTGGTTC	GTCCATATAGTTGAAGCAC	284
OSP	Sotub07g011080	XM_006344513	AT2G48030	CACCGCATATGAATTGTACCTCTC	CAGGGAGGGAGTTAACAAG	560
PED3	Sotub04g020700	XM_006355290	AT4G39850	CACCGTTAGTTCATCGCCATCCTCTG	GTACACCATTTAATTTTTGG	1127
PhDZnP	Sotub01g044570	XM_006346280	AT3G19510	CACCCTGAGATGTCAACTCTTGGAAAC	CAATCTTCAACCATTCACCG	2502
POD	Sotub02g027930	XM_015304568	AT5G66390	CACCTAAATCATGCCCACAAGCACAAG	CAGATGGAACAAGCGATG	1019
VAS	Sotub01g040060	XM_006349221	AT5G13900	CACCATTAATGGAGATGAAATAT	GATAGATCATACAAGGGAG	508
**qPCR**						
*qACT7**				GGAGAAGTTAGGTGGTGG	CAAACAACCTAAAAGAGACAC	181
*qBTH4**				GAAGAAGATAGGAAGGTGG	GGACATAAGAATCAACAAGCC	115
*qCCoAOMT1**				CTTGAACTTAATAGGTACATG	CTGCTTTAGAGGATTTATTATC	168
*qCFT**				CGATGATTTATGTTGTTATTTG	GATGTATACTGCTTTCTTCTC	147
*qGPAT3**				GGTGGGGTTCTTGGATTTG	TAACAGAATCCCCTCAAAGC	160
*qH2B**				CAGCTCTTAATCAGTTTTAG	CATTCAAGGAATAGCTATG	156
*qH3**				GTTACTATCATGCCTAAGG	GAATCGAGTAAATTCTGAATC	158
*qH4**				GGAAGACTGTTACTGCTATG	CTACTAATACCCAATAACC	109
*qMTN**				CCATCATCGAAGGAGTTGC	GTCCATATAGTTGAAGCAC	172
*qOSP**				CATCATTTACTAAAGATTTTG	CAGGGAGGGAGTTAACAAG	173
*qPED3**				GTACACCATTTAATTTTTGG	CATAATCTTTCCGGTCAGC	138

qPhDZnP				GACACTCCCCAAAGTAAGAC	CAATCTTCAACCATTCACCG	198
*qPOD**				CAGATGGAACAAGCGATG	GCCAAGTCTATGGTTAAGATGG	217
*qVAS**				GTCTTAAAGGTGCATCAAATTC	CAACAATTAATACACAAAGG	145
**Reference gene**						
qNAC	Sotub10g027110	XM_006339185	AT3G12390	ATATAGAGCTGGTGATGACT	TCCATGATAGCAGAGACTA	97
qUbq	Sotub12g030900	Y10024.1	AT2G47110	GTTTTACAAGGTTGATGATTC	GAGCAAAATCTAAGATAAAGC	202
qACT	Sotub10g022240	XM_006351284	AT3G12110	GAGTTGATTGTTGGGTTATG	GTGAAATGATTTAATGATGTG	179

Underlined CCAC was required for pENTER cloning
*Primers designed based on phellogen transcriptome sequences
